# Human Cardiac Organoids Reveal *HAND1* Loss-Associated Alterations in Cell Composition During Early Cardiac Differentiation

**DOI:** 10.3390/cells15211948

**Published:** 2026-10-26

**Authors:** Hao Yang, Yunqian Zeng, Yiyang Teng, Qi Zhao, Minbo Hou, Zhikai Cheng, Xuemei Zhang, Xijie Wang

**Affiliations:** 1 School of Pharmaceutical Sciences, Fudan University, Shanghai 201203, China; haoyang23@m.fudan.edu.cn; 2 China State Institute of Pharmaceutical Industry Co., Ltd., Shanghai 201203, China; 3 Shanghai InnoStar Bio-Tech Co., Ltd., Shanghai 201203, China; yqzeng@innostarbio.com (Y.Z.); yyteng@innostarbio.com (Y.T.); qzhao@innostarbio.com (Q.Z.); mbhou@innostarbio.com (M.H.); zkcheng@innostarbio.com (Z.C.); 4 Innovation Center for AI and Drug Discovery, School of Pharmacy, East China Normal University, Shanghai 200062, China

**Keywords:** human induced pluripotent stem cells, cardiac organoids, heart and neural crest derivatives expressed 1, cardiomyocyte differentiation

## Abstract

**Highlights:**

**What are the main findings?**

- A cardiac organoid platform derived from human induced pluripotent stem cells (hiPSCs) was established, linking tissue phenotypes with time-course bulk transcriptomics and day-9 single-cell profiles.
- *HAND1* loss was associated with lower cardiomyocyte proportions and relative enrichment of mesodermal/mesenchymal-like cells. Chromatin analyses supported HAND1 occupancy at *GATA4-*, *HCN4*-, and *TNNT2*-associated regions.

**What are the implications of the main findings?**

- Relating impaired cardiomyocyte formation to changes in cell composition provides a broader understanding of early cardiac differentiation abnormalities beyond individual marker expression.
- The identified HAND1-occupied regions provide specific candidates for functional studies of cardiac differentiation in a human three-dimensional model.

**Abstract:**

Understanding early cardiac developmental abnormalities requires linking tissue phenotypes to the cellular populations produced during differentiation. We established a cardiac organoid platform derived from human induced pluripotent stem cells (hiPSCs) and combined phenotypic assays, time-course and day-9 single-cell transcriptomics, and cleavage under targets and tagmentation (CUT&Tag) to investigate developmental perturbations. Organoids contained multiple cell populations and exhibited stage-dependent cardiac transcriptional programs. Thalidomide exposure altered morphology and reduced TNNT2-positive tissue area, with lower calcium transient amplitude at 0.01 μg/mL. RNA sequencing at 10 μg/mL identified downregulation of heart and neural crest derivatives expressed 1 (*HAND1*), prompting a separate genetic investigation. Across three paired differentiation experiments using one characterized *HAND1*-knockout clone, reduced cardiomyocyte representation accompanied relative enrichment of mesodermal/mesenchymal-like cells. Selected cardiac-associated expression abnormalities persisted at day 15. Expression and chromatin occupancy analyses identified candidate regulatory associations, with locus-specific quantitative PCR supporting HAND1 occupancy at selected cardiac-associated regions. Independent genetic and functional validation is needed to test causal relationships. Together, these findings place *HAND1* loss-associated tissue abnormalities in their cellular context and identify candidate occupied regions for investigating early cardiac differentiation in a human three-dimensional model.

## 1. Introduction

Early human cardiac development involves interconnected processes, including mesoderm induction, cardiac progenitor formation, differentiation of cardiomyocytes and non-cardiomyocytes, and tissue morphogenesis [1,2]. Cellular atlases of the developing human heart have further delineated stage-specific changes in cell populations and regional gene expression patterns [3,4]. Because access to early human embryonic tissues is limited, these processes are studied mainly in animal models and in vitro systems [5,6]. Human three-dimensional models that permit controlled perturbation are therefore important tools for investigating cardiac development and its abnormalities.

Human induced pluripotent stem cells (hiPSCs) provide an expandable cell source for human differentiation models [7]. Stage-specific signaling can guide hiPSC-derived cardiac organoids to form three-dimensional structures containing cardiomyocytes and other relevant cell populations, allowing cardiac differentiation and tissue self-organization to be studied [6,8,9,10]. These models include regionally patterned primitive heart structures, epicardial–myocardial organization, and the co-development of cardiac and hematopoietic tissues [11,12,13]. Such models and related three-dimensional cardiac systems have been used to investigate congenital developmental abnormalities [14], the effects of the maternal metabolic environment [15], and drug responses [16,17]. Assessing perturbations during differentiation requires examining whether cardiomyocyte formation and tissue composition change and whether established cardiomyocytes function normally. Combining morphological readouts with time-course and single-cell transcriptomics therefore characterizes perturbations relative to a normal-differentiation baseline.

Cardiac differentiation is jointly regulated by temporally coordinated signaling and transcription factor networks [18,19,20], and distinct signaling conditions have also been used to establish human multilineage heart field differentiation systems [21]. Heart and neural crest derivatives expressed 1 (HAND1) is a member of the basic helix–loop–helix (bHLH) transcription factor family and participates in cardiac developmental processes [22]. In mice, loss of *Hand1* can cause cardiac and extraembryonic abnormalities [23,24], while loss of myocardial HAND1 function can lead to structural and functional cardiac defects [25]. *Hand1* and *Hand2* gene dosage influences embryonic ventricular expansion [26]. HAND1 contributes to cavity morphogenesis in three-dimensional cardioid models [9]. Studies in human differentiation systems have revealed stage- and cell-population-dependent variation in *HAND1* and *HAND2* expression [27]; loss of *HAND1* alone can alter heart field-associated gene expression and cardiomyocyte subtype characteristics without necessarily preventing cardiomyocyte differentiation [28]. Further studies in three-dimensional differentiation systems have shown that HAND1 regulates progenitor fate in an expression level-dependent manner, with low and high expression levels supporting distinct mesodermal differentiation outcomes [29]. These findings suggest that HAND1 function must be interpreted in relation to its expression level, developmental stage, and differentiation context, rather than characterized simply as universally promoting cardiomyocyte differentiation.

Assessing HAND1 function therefore requires relating cardiomyocyte formation to the overall cellular composition produced during cardiac differentiation. Distinct mesodermal populations can give rise to different cardiomyocyte subtypes, while early mesoderm assembly is also associated with subsequent cell fate and tissue morphogenesis [30,31]. Studies of human cardiomyocyte differentiation have also shown that distinct differentiation states and transcriptional regulatory programs can coexist within the same culture system [32,33]. In multicellular cardiac organoids, altered cardiomyocyte-related phenotypes may involve changes in expression within cardiomyocytes, changes in progenitor-like or other non-cardiomyocyte populations, or both. We therefore examined which changes in cellular composition and developmental state accompany altered cardiomyocyte formation after *HAND1* loss, and how these changes relate to cardiac-associated regulatory programs.

Here, we optimized culture conditions to establish a reference for normal differentiation in human cardiac organoids. We then applied thalidomide as an exogenous perturbation, identifying *HAND1* as a candidate for genetic investigation. Using a *HAND1*-knockout hiPSC line, single-cell profiling, and cleavage under targets and tagmentation (CUT&Tag), we examined changes in cardiomyocyte formation, cellular composition, and candidate regulatory associations after *HAND1* loss.

## 2. Materials and Methods

### 2.1. hiPSCs Culture and Quality Control

The established parental hiPSC line was purchased from Beijing Cellapy Biotechnology Co., Ltd. (Beijing, China; Cellapy, CA4042106). hiPSCs were maintained in Essential 8 (E8) medium (Thermo Fisher Scientific, Waltham, MA, USA, A1517001) on six-well plates coated with Matrigel (Corning, Tewksbury, MA, USA, 354277) at 37 °C and 5% CO_2_, with daily medium changes. At approximately 70% confluence, cells were passaged using Gentle Cell Dissociation Reagent (STEMCELL Technologies, Vancouver, BC, Canada, 100-0485) and reseeded in E8 containing 10 μM Y-27632 (Selleck, Houston, TX, USA, S1049). Pluripotency markers, karyotype, and mycoplasma status were routinely assessed.

### 2.2. Cardiac Organoid Differentiation and Parameter Optimization

We followed a published differentiation protocol [6] and compared CHIR99021 concentrations. hiPSCs were dissociated into single cells, counted using an automated counter, and assessed by trypan blue exclusion (>90% viability). Under basal conditions, 10,000 cells were seeded in 100 μL E8 per well of U-shaped ultra-low attachment 96-well plates (Corning, 7007, Tewksbury, MA, USA).

On day −1, 50 μL of supernatant was gently removed from each well, and 200 μL of E8 medium without Y-27632 was added to bring the final volume to 250 μL. Day 0 was defined as the first CHIR99021 induction, when the medium was replaced with differentiation medium consisting of RPMI 1640 (Thermo Fisher Scientific, 11875085), B27 supplement without insulin (Thermo Fisher Scientific, A1895601), CHIR99021 (absin, Shanghai, China, abs810052), BMP4 (Novoprotein, Shanghai, China, CR93), and Activin A (Novoprotein, C687). Under basal differentiation conditions, the final concentrations of CHIR99021, BMP4, and Activin A were 6 μM, 1.25 ng/mL, and 1 ng/mL, respectively. On day 1, 166 μL of spent medium was removed and replaced with an equal volume of fresh RPMI 1640/B27 medium without insulin. On day 2, the same medium-change procedure was performed using RPMI 1640/B27 medium without insulin containing Wnt-C59 (absin, abs810639) at a final concentration of 2 μM. On day 4, 166 μL of spent medium was removed and replaced with an equal volume of fresh RPMI 1640/B27 medium without insulin. Organoids were then cultured for 48 h without medium change. On day 6, the medium was replaced with RPMI 1640 supplemented with B27 containing insulin (Thermo Fisher Scientific, 17504044), and on day 7 WNT signaling was transiently reactivated by removing 166 μL of medium from each well and adding 166 μL of RPMI 1640/B27 medium containing insulin and a final concentration of 2 μM CHIR99021 for 1 h. This medium was then removed and replaced with fresh RPMI 1640/B27 medium containing insulin. Thereafter, the medium was refreshed every 48 h until the designated experimental endpoint. For concentration optimization, CHIR99021 was applied on day 0 at 4, 6, 8, or 10 μM, with all other conditions unchanged. Subsequent experiments used 6 μM CHIR99021, the lower of the two concentrations (6 and 8 μM) supporting favorable TNNT2-positive tissue area fractions and cardiac-associated gene expression.

### 2.3. Thalidomide Treatment

Thalidomide (Sigma-Aldrich, St. Louis, MO, USA, T144; 0.01, 0.1, 1, 10, or 30 μg/mL) was added during the medium change on day −1, 24 h after seeding, and maintained until day 9 by replenishment at each medium change. Dimethyl sulfoxide (DMSO) stocks were diluted before use; all treatment and vehicle-control groups contained 0.1% (*v*/*v*) DMSO. Day-9 assessments included bright-field imaging, projected organoid area, CellTiter-Glo 3D luminescence, and TNNT2 immunofluorescence. Calcium-transient amplitude was assessed in the vehicle and 0.01 μg/mL groups, in which spontaneous signals remained measurable. Bulk RNA-seq compared 10 μg/mL thalidomide with vehicle to characterize the transcriptional response accompanying morphological and cardiomyocyte-associated changes.

### 2.4. Bright-Field Imaging and Area Quantification

Bright-field images were acquired at identical magnification using a CKX53 inverted microscope (Olympus, Tokyo, Japan). Projected organoid areas were measured in ImageJ/Fiji (ImageJ 1.54g; National Institutes of Health, Bethesda, MD, USA) from manually delineated regions of interest (ROIs), assisted by threshold-based segmentation and manual correction of irregular or shadowed boundaries. Identical criteria were used across groups.

### 2.5. Immunofluorescence and Quantification

Cells or organoids (≤8 organoids per 1.5-mL tube) were fixed in 4% paraformaldehyde for 30–45 min at room temperature, washed three times in PBS containing 20 mM glycine (5 min each), and blocked overnight at 4 °C with shaking in KGC3114 (KeyGEN, Nanjing, China) containing 0.5% Triton X-100. Primary antibodies (Table S1) were diluted in KGC3115 containing 0.5% Triton X-100 and incubated overnight at 4 °C. After three DPBS washes, samples were incubated with fluorescent secondary antibodies in KGC3117 overnight at 4 °C in the dark, counterstained with DAPI (Solarbio, Beijing, China, C0065) for 1 h at room temperature, and washed in DPBS. Images were acquired using a spinning-disk confocal microscope (Olympus, Tokyo, Japan) with matched laser intensity, exposure, and gain, with z-spacing automatically determined by the imaging system. Maximum-intensity projections of at least three z-planes per organoid were analyzed in ImageJ/Fiji. Tissue ROIs were automatically defined from the overall DAPI-positive tissue contour. A TNNT2 threshold determined from controls was applied uniformly across groups within each experiment. The TNNT2-positive tissue area fraction was calculated as the threshold-positive area within the ROI divided by the total tissue ROI area. Projected tissue area fractions within the sampled optical planes are reported as percentages for CHIR99021 and CON/KO comparisons, or relative to vehicle for thalidomide (CON = 1).

### 2.6. TNNT2 Flow Cytometry

Day-9 organoids were dissociated into single-cell suspensions, centrifuged at 350× *g* for 3 min, and washed once in PBS. Cells were incubated with Aqua-BV510 viability dye for 30 min at 4 °C in the dark, washed, and fixed/permeabilized with 200 μL Fixation and Permeabilization Solution (BD, Franklin Lakes, NJ, USA, 554714) for 20 min at 4 °C. Intracellular TNNT2 was stained with Alexa Fluor 647-conjugated anti-cardiac troponin T (Abcam, Cambridge, UK, EPR20266; 1:500) for 30 min at 4 °C in the dark. Cells were washed in 1× washing buffer, resuspended in 300 μL buffer, and acquired on a BD LSRFortessa X-20 (Franklin Lakes, NJ, USA). Cells were sequentially gated by FSC-A/SSC-A, FSC-A/FSC-H, and low Aqua fluorescence in BV510-A to select cells, singlets, and viable singlets, respectively. TNNT2 positivity was assessed in APC-A and reported as the percentage of viable singlets. Representative gating is shown in Figure S1A.

### 2.7. ATP-Dependent Luminescence Assay

ATP-dependent luminescence on day 9 was measured using CellTiter-Glo 3D (Promega, Madison, WI, USA, G9682). Organoids in opaque-walled 96-well plates were equilibrated at 22–25 °C for approximately 30 min, mixed with 100 μL reagent per well, and shaken vigorously for 5 min. Luminescence was read on a SpectraMax i3x (Molecular Devices, San Jose, CA, USA; 1 s/well). Luminescence was corrected by subtracting cell-free blank signals and reported in relative light units (RLU), without normalization to vehicle controls.

### 2.8. Calcium Transient Imaging

Organoids were washed twice in RPMI 1640, loaded with 5 μM Fluo-4 AM (Thermo Fisher Scientific, F14201) for 30 min at 37 °C in the dark, and washed three times in RPMI 1640/B27. Confocal fluorescence recordings were acquired at ≥10 frames/s.

Time-series fluorescence (F) was extracted in ImageJ/Fiji from an ROI covering the main beating or fluorescence-changing region. Baseline fluorescence (F_0_) was estimated from each fluorescence trace using asymmetric least-squares smoothing [6]. Normalized fluorescence changes were calculated as ΔF/F_0_ = (F − F_0_)/F_0_. Transient amplitude for each organoid was calculated as the mean of the identified peak ΔF/F_0_ values. Representative fluorescence traces were displayed as F/F_median_, where F_median_ is the median ROI fluorescence over the displayed interval. No frequency analysis was performed on these traces. Recordings were excluded for marked focal drift, staining failure, or severe motion artifacts, but not solely for weak or nonperiodic signals.

### 2.9. Quantitative Real-Time PCR

Organoids were collected on days 0, 3, 9, 15, 21, and 30. Total RNA was extracted using Super FastPure Cell RNA Isolation Kit (Vazyme, Nanjing, China), quantified with NanoDrop One (Thermo Fisher Scientific), and reverse-transcribed using HisyGo All-in-One RT Red SuperMix for qPCR (+gDNA Wiper; Vazyme). cDNA was stored at −20 °C. Reactions (20 μL) used SYBR Green Master Mix (Vazyme) on a QuantStudio 7 Flex (Thermo Fisher Scientific). Relative expression was calculated using the 2^−ΔΔCt^ method with *GAPDH* as the reference gene and the calibrator specified in the corresponding figure legend. Primer sequences are listed in Table S2. Experiments included three independent biological replicates unless specified otherwise.

### 2.10. Generation and Validation of HAND1-KO hiPSCs

Cyagen Biosciences generated the *HAND1*-KO hiPSC line by electroporating CRISPR-Cas9 ribonucleoprotein complexes. Cell pools were selected by editing efficiency, followed by single-clone isolation, expansion, and Sanger genotyping. The *HAND1* sgRNA protospacer was AGCGCGAGGCCGGACCGAAG (PAM: AGG).

Five CCTop-predicted off-target sites (https://cctop.cos.uni-heidelberg.de/; accessed on 20 October 2026) were examined by PCR and Sanger sequencing; no obvious mutations were detected. Sequencing primers are listed in Table S3. All KO-versus-control organoid experiments used a single genotyped *HAND1*-KO clone and its parental line, with independently performed differentiation batches providing biological replication. Control and *HAND1*-KO hiPSCs were washed in cold PBS and lysed in RIPA buffer (Solarbio, R0020). Protein concentrations were measured by BCA assay. Equal protein amounts were separated by SDS-PAGE, transferred to PVDF membranes, blocked, and probed with anti-HAND1 and loading-control antibodies, followed by HRP-conjugated secondary antibodies (Table S1). Bands were detected by chemiluminescence.

### 2.11. Bulk RNA Sequencing

#### 2.11.1. Sample Preparation and Sequencing

Bulk RNA-seq included differentiation days 0, 3, 9, and 15, and day-9 organoids exposed to 10 μg/mL thalidomide or vehicle, with three independent biological replicates per group/time point. Total RNA was extracted using Super FastPure Cell RNA Isolation Kit (Vazyme). Genewiz (Suzhou, China) prepared libraries from 1 μg RNA per sample and performed sequencing. Analyses were performed in R using RStudio 2026.01.1+403 (Windows) and DESeq2 v1.50.2. Both nominal *p* values and false discovery rate (FDR)-adjusted *p*-values were reported; the differential-expression thresholds were |log_2_ fold change| ≥ 1 and FDR < 0.05. Heatmaps used normalized expression matrices. Functional enrichment analyses in R assessed biological processes and signaling pathways.

#### 2.11.2. Data Processing and Enrichment Analysis

FASTQ reads were processed with fastp v0.24.1 (Phred threshold, 20; maximum adapter-trimming mismatch rate, 10%; minimum length, 75 bp; maximum ambiguous bases, 5) and assessed with FastQC v0.11.9. Clean reads were aligned to GRCh38/hg38 using HISAT2 v2.2.1; samples with >70% alignment were retained. Gene counts were obtained with HTSeq v0.6.1.

### 2.12. Single-Cell RNA Sequencing Analysis

Day-9 scRNA-seq data from three batch-paired biological samples per group were processed with Cell Ranger v9.0.1 and Seurat v5.1.0. Cells with <200 detected genes, <500 UMIs, or >20% mitochondrial transcripts and genes detected in <3 cells were excluded. Predicted doublets were removed per sample using DoubletFinder v2.0.4. Data were log-normalized (scale factor, 10,000), and 2000 VST-selected variable genes were scaled for PCA. Harmony v1.2.3 (IntegrateLayers) was used for integration; clustering used 30 principal components and resolution 0.8, with UMAP and t-SNE visualization. Marker-based annotation used exploratory FindMarkers/FindAllMarkers comparisons (|log_2_ fold change| > 0.25; adjusted *p* < 0.05). Sample-level retention and quality metrics are provided in Table S5.

Raw UMI counts from all retained cells were summed per biological sample for DESeq2 v1.50.2 (~pair + group; CON reference). Genes with ≥10 counts in ≥3 samples were tested using Wald tests with Benjamini–Hochberg correction and independent filtering disabled. Candidates required FDR < 0.05 and |log_2_ fold change| ≥ 1. This all-cell analysis retains cell-composition effects.

Cell proportions used all retained cells per sample as the denominator. Exploratory comparisons used two-sided exact paired sign-flip tests of mean KO-minus-CON differences in percentage points across three differentiation batches, enumerating all eight sign assignments, with Holm correction across 10 categories (Table S6).

For Figure S8, 20,501 cells from original clusters 1, 3, 4, 15, 17, and 24 were reanalyzed without condition balancing using R v4.5.3 and Seurat v5.4.0. Variable genes were selected anew, independently of the original marker panel. Leiden clustering based on 30 uncorrected principal components was evaluated across resolutions and seeds. The lowest multicluster resolution meeting median adjusted Rand index ≥ 0.90 and median bidirectional best-match Jaccard index ≥ 0.75 across seeds was selected (resolution 0.2; seed 11). Sensitivity analyses included paired-batch Harmony v2.0.5, leave-one-KO-sample-out reanalysis, quality-metric filtering, and cell-cycle assessment. Quality-filtering checks did not alter the reported composition counts. State markers were evaluated descriptively within samples using sample–state aggregates. Pools containing ≥ 20 cells were compared with Tyser et al.’s human Carnegie stage 7 reference [34] by Spearman correlation using 2000 reference-selected variable genes. Detailed clustering, marker-selection, quality-control, and reference-comparison procedures are provided in the Supplementary Methods.

### 2.13. CUT&Tag Assay

HAND1 chromatin occupancy was profiled using CUT&Tag [35]. Day-9 organoid cells were washed twice and bound to ConA beads (10 μL; Bangs Laboratories, Fishers, IN, USA) for 10 min at room temperature, incubated with mouse anti-HAND1 (Abcam, Cambridge, UK, ab279390; 1:50) overnight at 4 °C, and then with goat anti-mouse IgG (N270; 1:100) for 1 h at room temperature. After 2–3 digitonin-buffer washes, cells were incubated with pA–Tn5 (1:100) for 1 h at room temperature, washed, and tagmented in 10 mM MgCl_2_ for 1 h at 37 °C. Reactions were stopped with SDS at 55 °C for 10 min. DNA was extracted using Tagment DNA Extract Beads, amplified using NEBNext HiFi 2× PCR Master Mix, bead-purified, and assessed using a 4200 TapeStation (Agilent Technologies, Santa Clara, CA, USA). Shanghai Jiayin Biotechnology (Shanghai, China) performed library preparation and paired-end 150-bp sequencing on a NovaSeq 6000 platform (Illumina, San Diego, CA, USA).

Quality-filtered reads were aligned using BWA, and uniquely mapped reads were used for MACS2 peak calling (*q* < 0.05). BEDTools assigned genomic features using ≥ 1-bp overlap, prioritizing promoter bins within 1 kb, 1–2 kb, and 2–3 kb of the TSS on either side, followed by 5′ UTR, 3′ UTR, exon, intron, and intergenic regions. Shared peak centers were associated with the nearest Ensembl transcript TSS within 100 kb. Tied gene assignments were split and gene symbols deduplicated before descriptive intersection with pseudobulk results. Read-processing, motif, enrichment, and differential-peak analysis details are provided in the Supplementary Methods.

### 2.14. CUT&Tag-qPCR

Regions associated with GATA4, MYOCD, HCN4, TNNT2, TBX5, and WNT5B were selected using HAND1 CUT&Tag peak support, expression data, and developmental relevance, without restriction to promoters. The six candidate amplicons and NEG were mapped in silico to GRCh38/hg38; predicted product sizes were 80–129 bp, and qPCR products were not sequenced. Primer sequences, amplicon coordinates, transcript-relative locations, and replicate-specific peak support are reported in Table S4 using 1-based inclusive coordinates.

NEG (chr5:21,299,800–21,300,200) was selected from the same dataset because no peak was called in any of the three replicates. It served as the within-sample genomic reference and was not independently validated as unoccupied; IgG controlled antibody-associated background.

Three independent biological samples were analyzed with matched HAND1 and IgG measurements, each in technical triplicate. Technical Ct values were averaged before calculating ΔCt = Ct(target) − Ct(NEG) separately for each antibody condition. Signals were displayed as 2^−ΔCt^ (mean ± SEM), and HAND1–IgG comparisons used paired *t*-tests on biological-sample ΔCt values (*n* = 3 pairs).

### 2.15. Statistical Analysis and Replication

Statistical analyses used GraphPad Prism 10.1.0 for Windows (GraphPad Software, Boston, MA, USA) unless otherwise specified. Data are presented as mean ± SEM from three independent differentiation experiments unless otherwise stated. For organoid-level measurements, the mean per group within each experiment was used as one biological replicate. Unless otherwise specified in Section 2.11, Section 2.12, Section 2.13 and Section 2.14, two-group comparisons used two-tailed unpaired Student’s t-tests, and multiple-group comparisons used one-way ANOVA followed by Tukey’s or Dunnett’s multiple-comparison tests. Sample sizes are specified in the corresponding figure legends. UMAPs and cell-level expression distributions were descriptive. Significance was denoted as * *p* < 0.05, ** *p* < 0.01, *** *p* < 0.001, **** *p* < 0.0001; ns, *p* ≥ 0.05.

## 3. Results

### 3.1. Establishment and Optimization of a Human Cardiac Organoid Differentiation System

**Figure 1:**
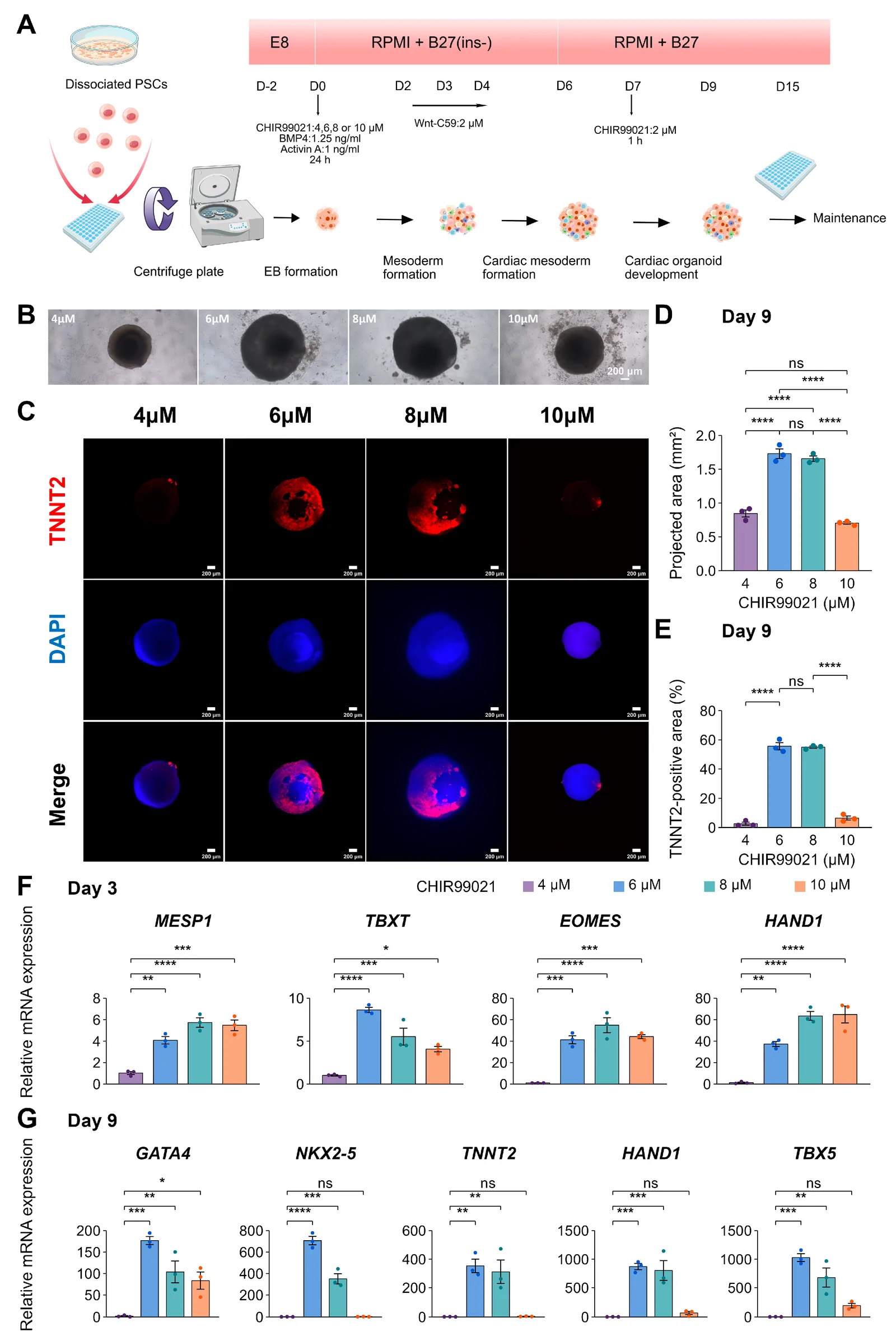
Optimization of hiPSC-derived cardiac organoid differentiation. (**A**) Differentiation scheme and CHIR99021 optimization, created with GDP [38]. (**B**,**C**) Representative day-9 bright-field and TNNT2 immunofluorescence images after induction with 4, 6, 8, or 10 μM CHIR99021. TNNT2, red; DAPI, blue; scale bars in (**B**,**C**), 200 μm. (**D**) Projected organoid area (mm^2^); six organoids per group per experiment. (**E**) TNNT2-positive tissue area fraction (%), calculated within the tissue ROI; three to four organoids per group per experiment. (**F**) Day-3 RT-qPCR analysis of *MESP1*, *TBXT*, *EOMES*, and *HAND1*. (**G**) Day-9 RT-qPCR analysis of *GATA4*, *NKX2-5*, *TNNT2*, *HAND1*, and *TBX5*. Data are mean ± SEM from three independent experiments; for organoid measurements, each experiment’s mean is one replicate. Brackets indicate group comparisons using one-way ANOVA. * *p* < 0.05, ** *p* < 0.01, *** *p* < 0.001, **** *p* < 0.0001; ns, *p* ≥ 0.05.

We optimized the CHIR99021 concentration used for initial induction in a previously reported cardiac organoid differentiation protocol [6]. Figure 1A outlines the differentiation workflow. We compared organoid formation following treatment with 4, 6, 8, or 10 μM CHIR99021. Bright-field imaging revealed differences in organoid size across conditions, with larger organoids observed in the 6 and 8 μM groups (Figure 1B). Quantification of organoid projected area showed significantly greater areas in both the 6 and 8 μM groups than in the 4 and 10 μM groups, with no statistically significant difference detected between the 6 and 8 μM groups (Figure 1D). To assess cardiomyocyte differentiation under these conditions, we performed TNNT2 immunofluorescence staining on differentiation day 9. TNNT2-positive regions were more extensive in the 6 and 8 μM groups than in the 4 and 10 μM groups, and the TNNT2-positive area fraction was significantly higher in the 6 and 8 μM groups than in the 4 μM group (Figure 1C,E); no statistically significant difference was detected between the 6 and 8 μM groups (Figure 1E). Further qPCR analysis showed that, on differentiation day 3, expression of the mesoderm-associated genes *MESP1*, *TBXT*, and *EOMES*, as well as *HAND1*, was higher in the 6, 8, and 10 μM groups than in the 4 μM group, although concentration–response patterns varied across genes (Figure 1F). On differentiation day 9, expression of *GATA4*, *NKX2-5*, *TNNT2*, *HAND1*, and *TBX5* was significantly higher in both the 6 and 8 μM groups than in the 4 μM group; by contrast, differences between the 10 and 4 μM groups did not reach statistical significance for any of these genes except *GATA4* (Figure 1G). Considering organoid projected area, TNNT2-positive tissue area fraction, and cardiac-associated gene expression, we selected 6 μM CHIR99021, the lower of the two concentrations supporting favorable differentiation outcomes, for subsequent experiments.

### 3.2. Composition and Transcriptional Dynamics of Human Cardiac Organoids

**Figure 2:**
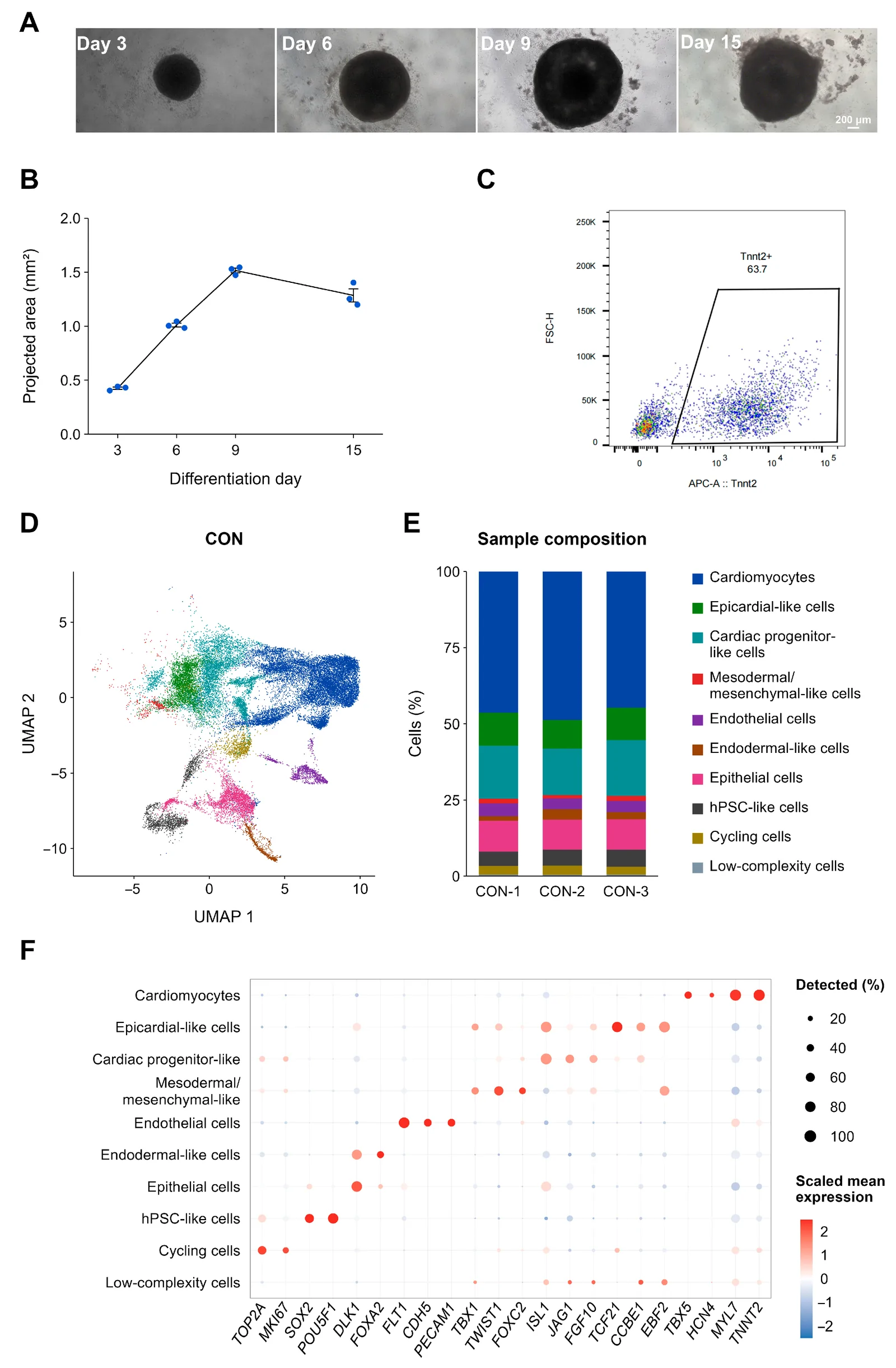
Morphology and cellular composition of cardiac organoids. (**A**) Bright-field images on days 3, 6, 9, and 15; scale bar, 200 μm. (**B**) Projected organoid area (mm^2^) across differentiation days. Points represent the means of eight organoids per time point in each of three independent differentiation experiments; data are mean ± SEM. (**C**) Representative day-9 flow cytometry: 63.7% TNNT2-positive viable singlets; colors indicate event density (blue, low; red, high). Across three independent differentiations, the mean was 59.3% (range, 56.7–63.7%); complete gating is shown in Figure S1A. (**D**) UMAP of 31,510 CON cells in the shared CON/KO embedding; colors identify annotated cell populations (key in E). (**E**) Cell composition per CON sample, calculated relative to all retained cells in that sample. (**F**) Marker expression in the annotated CON populations: dot size indicates transcript-detection frequency and color indicates scaled mean expression.

Using the optimized differentiation conditions, we maintained the organoids in culture until day 15. Bright-field imaging and area quantification showed that organoid projected area was smaller on day 15 than on day 9 (Figure 2A,B). To assess the cardiomyocyte content of the organoids, we performed flow cytometry on differentiation day 9. Across three independent differentiation experiments, the proportion of TNNT2-positive cells ranged from 56.7% to 63.7%, with a mean of 59.3% (Figure 2C). Marker-based annotation of day-9 control scRNA-seq data identified cardiomyocytes, cardiac progenitor-like, epicardial-like, endothelial, and mesodermal/mesenchymal-like cells, together with epithelial and endodermal-like populations (Figure 2D,F). Published organoid and endoderm studies provided context for marker interpretation [36,37]. Relative cell proportions are shown separately for the three CON samples in Figure 2E, with complete numerical summaries in Table S6.

**Figure 3:**
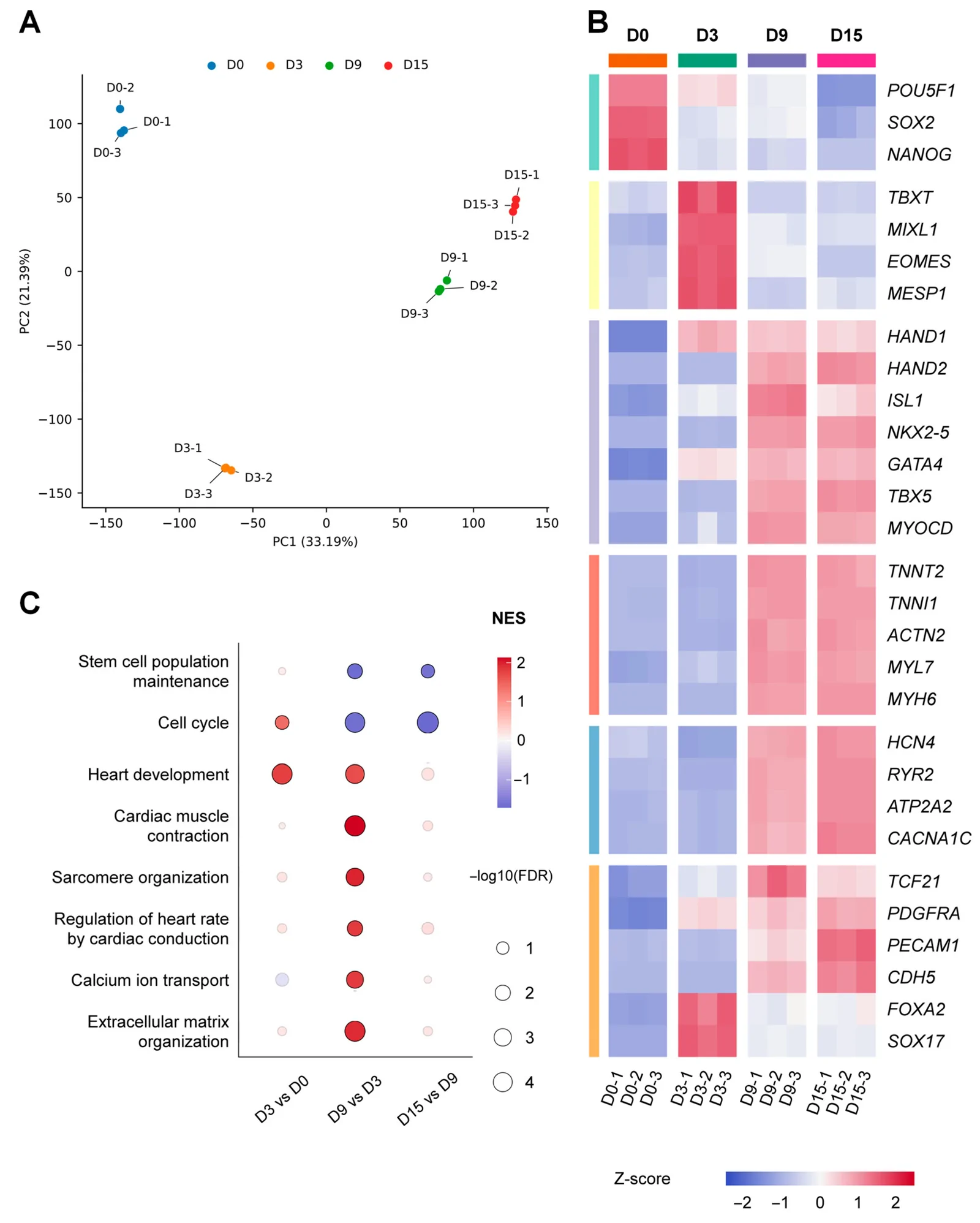
Transcriptional dynamics during organoid differentiation. (**A**) PCA of day-0, -3, -9, and -15 samples, with three independent differentiation samples per time point. Each point represents one sample; axes indicate the proportion of variance explained. (**B**) Heatmap of developmental gene expression. Columns represent individual samples, with three independent differentiation samples per time point. Expression values were log_2_(FPKM + 1)-transformed and Z-score-standardized per gene across all 12 samples; blue and red indicate relatively lower and higher expression, respectively. The left annotation bar identifies six functional gene groups, ordered from top to bottom: pluripotency-associated genes (turquoise), primitive streak/mesoderm-associated genes (pale yellow), cardiac transcription factors (lavender), cardiomyocyte structural genes (coral), electrophysiology/calcium-handling genes (light blue), and non-cardiomyocyte-associated genes (light orange). The top annotation bars indicate D0 (orange), D3 (green), D9 (purple), and D15 (magenta). (**C**) GSEA summary for D3 versus D0, D9 versus D3, and D15 versus D9 using signed differential-expression statistics. Color indicates the normalized enrichment score (NES), and dot size indicates −log_10_(FDR). Positive and negative NES denote enrichment toward higher expression at the later and earlier stages, respectively. Descriptive gene-expression trends and representative enrichment curves are shown in Figure S1B–E. Gene sets were derived from GO Biological Process annotations. FDR values were calculated by Benjamini–Hochberg correction across all 1712 tested gene sets within each comparison; FDR < 0.05 was considered significant.

To characterize transcriptomic changes during organoid differentiation, we performed RNA sequencing on samples collected on days 0, 3, 9, and 15. In principal component analysis, samples separated by differentiation stage, and replicates from the same time point clustered relatively closely (Figure 3A). Marker gene analysis showed an overall decrease in the expression of the pluripotency-associated genes *POU5F1*, *SOX2*, and *NANOG* [28] following differentiation, whereas the early mesoderm-associated genes *TBXT*, *MIXL1*, *EOMES*, and *MESP1* [28,39] showed relatively high expression on day 3. Expression of the cardiac-associated transcription factors *NKX2-5*, *GATA4*, and *TBX5* [40] and the cardiomyocyte structural genes *TNNT2*, *ACTN2*, and *MYH6* [41] varied across differentiation stages. Several cardiomyocyte-associated genes remained relatively highly expressed on days 9 and 15, supporting the establishment of cardiomyocyte differentiation programs (Figure 3B and Figure S1B). The heatmap in Figure 3B shows gene-wise Z-scores derived from sample-level log_2_(FPKM + 1) values. The independently rescaled curves in Figure S1B describe within-gene temporal patterns only; equal plotted heights do not imply equal transcript abundance. Subsets of extracellular matrix/mesenchymal-associated genes [42] and endothelial-associated genes [43] also changed over time, which is consistent with temporal shifts in stromal- and endothelial-associated expression programs. Gene set enrichment analysis (GSEA) [44] further delineated changes in functional programs between adjacent differentiation stages. Compared with day 0, day 3 samples showed positive enrichment of gene sets related to heart development. The transition from day 3 to day 9 was characterized primarily by positive enrichment of gene sets related to cardiac muscle contraction, sarcomere organization, calcium ion transport, and cardiac conduction, together with negative enrichment of cell-cycle and stem-cell population maintenance gene sets. From day 9 to day 15, cell-cycle-related gene sets remained negatively enriched, whereas most selected gene sets related to cardiac function did not show statistically significant enrichment (Figure 3C and Figure S1C–E). Overall, the cellular and time-course analyses establish a multicellular differentiation reference in which cardiomyocyte-associated programs are evident by day 9, providing a basis for interpreting perturbation-associated changes.

### 3.3. Thalidomide Exposure Induces Phenotypic and Transcriptional Changes in Cardiac Organoids

**Figure 4:**
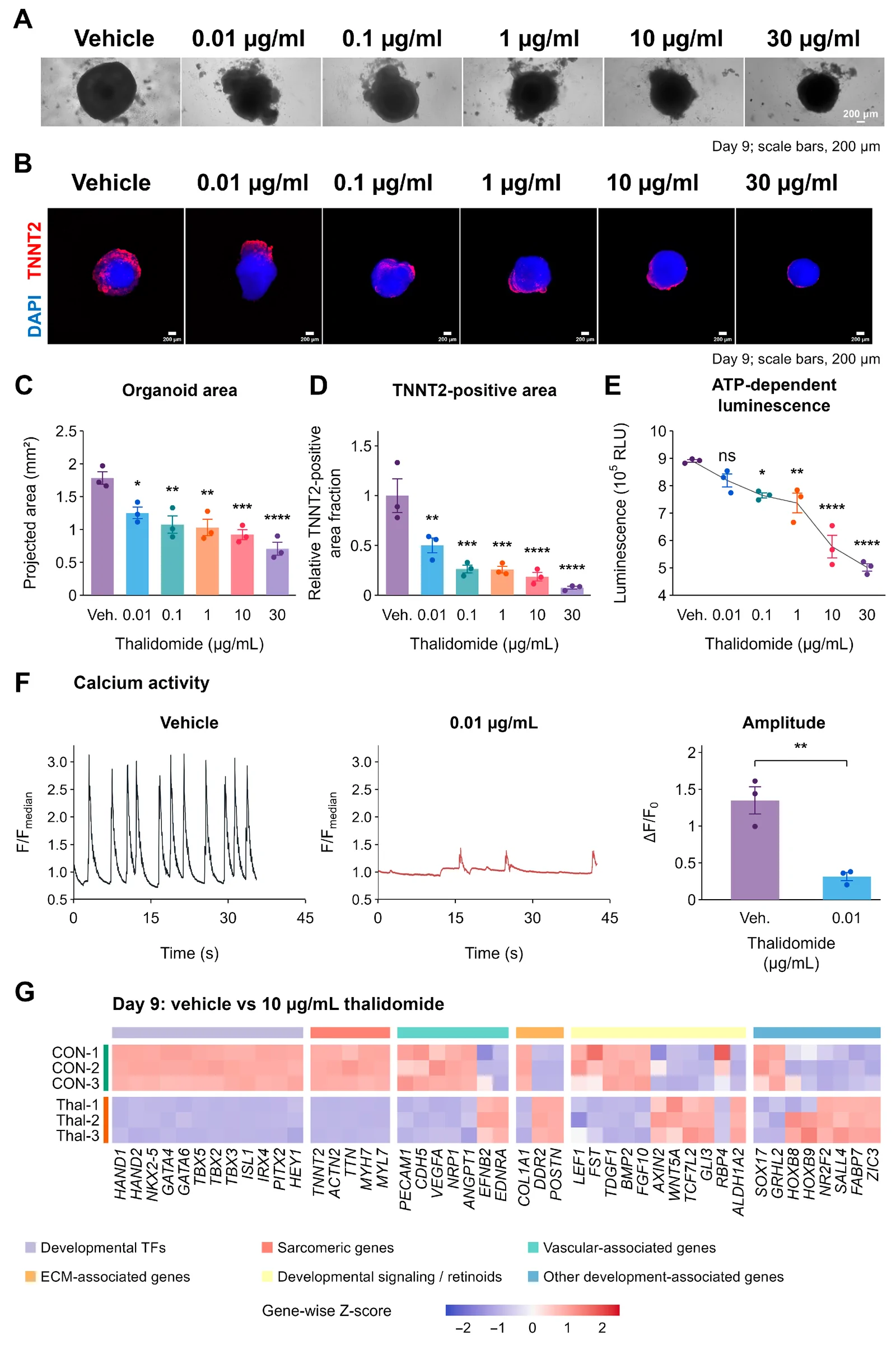
Effects of thalidomide exposure on cardiac organoids. (**A**,**B**) Representative day-9 bright-field (**A**) and TNNT2 immunofluorescence (**B**) images following continuous exposure to vehicle or 0.01, 0.1, 1, 10, or 30 μg/mL thalidomide. TNNT2, red; DAPI, blue; scale bars, 200 μm. (**C**) Projected organoid area (mm^2^). (**D**) TNNT2-positive tissue area fraction normalized to vehicle (CON = 1). (**E**) CellTiter-Glo 3D luminescence, expressed in 10^5^ relative light units (RLU). (**F**) Representative spontaneous calcium traces, displayed as F/F_median_, and calcium-transient amplitude (ΔF/F_0_) in vehicle and 0.01 μg/mL thalidomide groups. For (**C**–**E**), and the amplitude analysis in F, respectively, 9, 3, 5, and 4 organoids were measured per group per experiment; each point represents the corresponding experiment-level mean. Data are mean ± SEM from three independent experiments. (**G**) Heatmap of representative genes from bulk RNA-seq of day-9 organoids treated with 10 μg/mL thalidomide or vehicle, with three independent biological replicates per group. Rows represent samples and columns represent genes grouped as developmental transcription factors, sarcomeric genes, vascular-associated genes, extracellular matrix-associated genes, developmental signaling/retinoid-related genes, and other development-associated genes. Colors indicate gene-wise Z-scores. The corresponding volcano plot is shown in Figure S2B. * *p* < 0.05, ** *p* < 0.01, *** *p* < 0.001, **** *p* < 0.0001; ns, *p* ≥ 0.05.

We next evaluated the response to exogenous developmental perturbation. Using thalidomide, a compound with well-documented teratogenic effects [45], as a model perturbagen, we continuously exposed organoids from day −1 and assessed their phenotypes on day 9. Bright-field imaging revealed varying degrees of morphological change in thalidomide-treated organoids compared with vehicle controls, including irregular boundaries in some samples (Figure 4A). Quantification of projected area showed smaller organoids in the treatment groups, with an overall decreasing trend as drug concentration increased (Figure 4C). CellTiter-Glo 3D measurements likewise showed decreasing ATP-dependent luminescence with increasing concentration, although the difference between the 0.01 μg/mL group and the control group did not reach statistical significance (Figure 4E). At this concentration, organoid projected area was already significantly reduced, indicating that morphological measurements provide information on drug responses beyond that captured by the ATP-dependent assay. TNNT2-positive tissue area also decreased across thalidomide concentrations (Figure 4B,D). In addition, the amplitude of spontaneous calcium transients, expressed as ΔF/F_0_, was significantly lower in the 0.01 μg/mL thalidomide group than in controls, and representative calcium traces further illustrated differences in calcium signals between the groups (Figure 4F and Figure S2A). Calcium-transient analysis was limited to 0.01 μg/mL because reproducible transients could not be reliably resolved at higher tested concentrations under the recording conditions. Bulk RNA-seq at 10 μg/mL was used to characterize the transcriptional state associated with the more pronounced morphological and cardiomyocyte-related phenotype. Differential expression analysis using DESeq2 [46] identified downregulation of cardiac-associated genes, including *HAND1*, *TBX20*, *TBX5*, *NKX2-5*, and *TNNT2* (Figure S2B). A heatmap of representative genes showed lower expression of several cardiac-associated transcription factors and cardiomyocyte structural genes, but higher expression of some development-associated genes, in the treatment group (Figure 4G). Among the downregulated cardiac-associated genes, *HAND1* was selected for subsequent genetic investigation because of its established role in early cardiac development.

### 3.4. HAND1 Loss Is Associated with Altered Cardiac Differentiation and Cellular Composition

To investigate the role of HAND1 in cardiac organoid differentiation, we generated and characterized a *HAND1*-knockout hiPSC line. Target-site sequencing, analysis of the selected predicted off-target sites, RT-qPCR, and Western blotting were used to verify gene editing and changes in HAND1 expression (Figure S2C–E).

**Figure 5:**
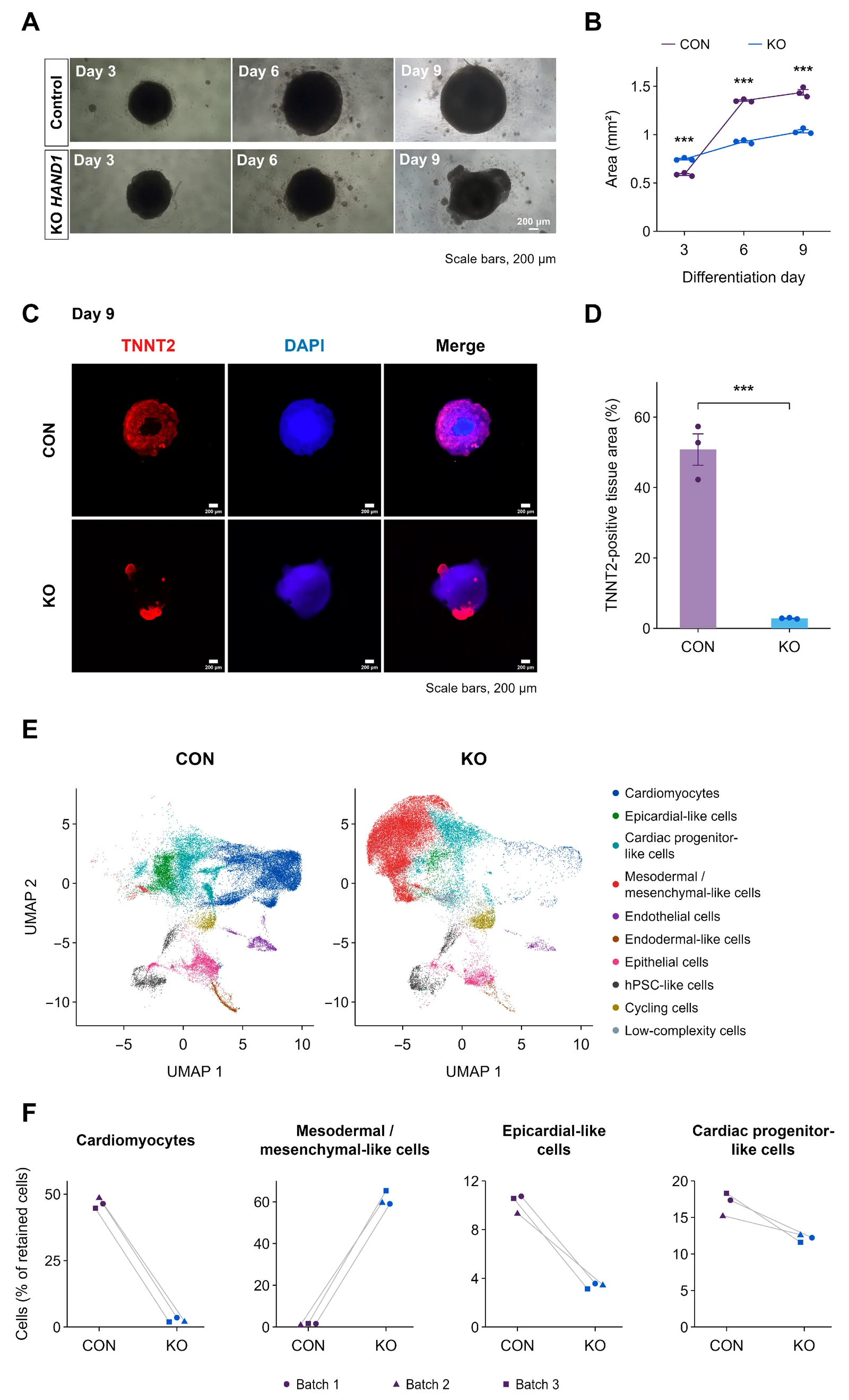
*HAND1* loss-associated differentiation and cell-composition changes. (**A**,**B**) Representative bright-field images (**A**) and projected organoid areas (**B**) on days 3, 6, and 9, with six organoids per group per experiment. (**C**,**D**) Representative day-9 TNNT2 immunofluorescence images (**C**) and TNNT2-positive tissue area fraction (%, D), calculated relative to the total tissue ROI area, with five organoids per group per experiment. TNNT2, red; DAPI, blue; scale bars in (**A**,**C**), 200 μm. Quantification in (**B**,**D**) is shown as mean ± SEM from three independent differentiation experiments; each point represents the experiment-level organoid mean. (**E**) Condition-split UMAP of 63,954 retained day-9 cells from three paired CON/KO differentiation experiments, displayed in a shared integrated embedding and colored by cell annotation. (**F**) Sample-level proportions of cardiomyocytes, mesodermal/mesenchymal-like cells, epicardial-like cells, and cardiac progenitor-like cells, using all retained cells in each sample as the denominator. Symbols identify the three paired differentiation batches, with lines connecting matched CON and KO samples. *HAND1* expression, developmental-gene RT-qPCR, and full sample-composition profiles are shown in Figure S3. *** *p* < 0.001. (**F**) presents paired cell proportions; corresponding sample-level statistics and exploratory paired tests are provided in Table S6.

Bright-field imaging revealed morphological differences between control and *HAND1*-KO organoids on days 3, 6, and 9. Area quantification showed that organoid projected area was lower in the KO group than in controls on days 6 and 9 (Figure 5A,B). During normal differentiation, RT-qPCR showed stage-dependent *HAND1* expression that was highest on day 3 among the time points examined and gradually declined thereafter (Figure S3A). On day 3, RT-qPCR showed higher expression of *MIXL1*, *TBXT*, and *HOXB1* in the *HAND1*-KO group than in controls, whereas *MESP1* expression did not differ significantly (Figure S3C). Thus, selected developmental expression differences were already detectable by day 3 without a broad reduction in the early mesoderm-associated genes examined. TNNT2 immunofluorescence staining on day 9 further showed a significantly lower TNNT2-positive area fraction in KO organoids than in controls, suggesting impaired formation of cardiomyocyte-containing tissue (Figure 5C,D).

We used single-cell RNA sequencing to examine the cellular composition of day 9 control and *HAND1*-KO organoids, with three independent samples per group paired by differentiation batch. The dataset used for the present analysis comprised 63,954 cells, including 31,510 control cells and 32,444 KO cells, distributed across 24 retained clusters. Based on marker gene expression, cells were annotated as cardiomyocytes, epicardial-like cells, cardiac progenitor-like cells, mesodermal/mesenchymal-like cells, endothelial cells, endoderm-like cells, epithelial cells, and human pluripotent stem cell (hPSC)-like cells, with proliferative states and low-complexity cells annotated separately. UMAP visualization in the shared integrated embedding revealed distinct distributions of control and KO cells (Figure 5E), and the distribution of *HAND1* transcripts in these cells is shown in Figure S3B.

Sample-level composition analysis showed markedly lower cardiomyocyte proportions in *HAND1*-KO organoids (CON, 46.64 ± 2.03%; KO, 2.52 ± 0.89%) and relative enrichment of mesodermal/mesenchymal-like cells (CON, 1.46 ± 0.29%; KO, 61.37 ± 3.46%). The mean paired KO-minus-CON differences were −44.13 and +59.91 percentage points, respectively, with the same direction of change across all three differentiation batches. Epicardial-like and cardiac progenitor-like proportions were also lower in KO samples (Figure 5F and Figure S3F; Table S6). Exploratory exact paired tests did not reach statistical significance after Holm adjustment. Whole-organoid RT-qPCR further showed lower expression of *TNNT2*, *GATA4*, and *TBX5* and higher expression of *FOXC2*, *TBX1*, *HOXA1*, and *NR2F2* in the KO group on day 9, whereas the difference in *ISL1* expression did not reach statistical significance (Figure S3D). On day 15, expression of *TNNT2*, *NKX2-5*, *TBX5*, and *MYL7* remained lower in the KO group than in controls, while *TBX1* and *NR2F2* expression remained higher, and increased *FGF8* and *FGF10* expression was also observed (Figure S3E).

Calcium recordings were obtained in three independent CON/KO differentiation experiments. One representative matched pair is presented in Figure S4; the displayed KO trace lacks the clear repetitive fluctuations seen in the CON trace during the shown interval. No pooled amplitude or frequency comparison is presented. Taken together, reduced TNNT2-positive tissue and directionally consistent cell-composition differences characterize altered differentiation in this *HAND1*-knockout model, with selected cardiac-associated expression abnormalities still detectable at day 15.

### 3.5. Transcriptional Features of KO-Enriched Cells and HAND1 Chromatin Occupancy at Cardiac-Associated Loci

**Figure 6:**
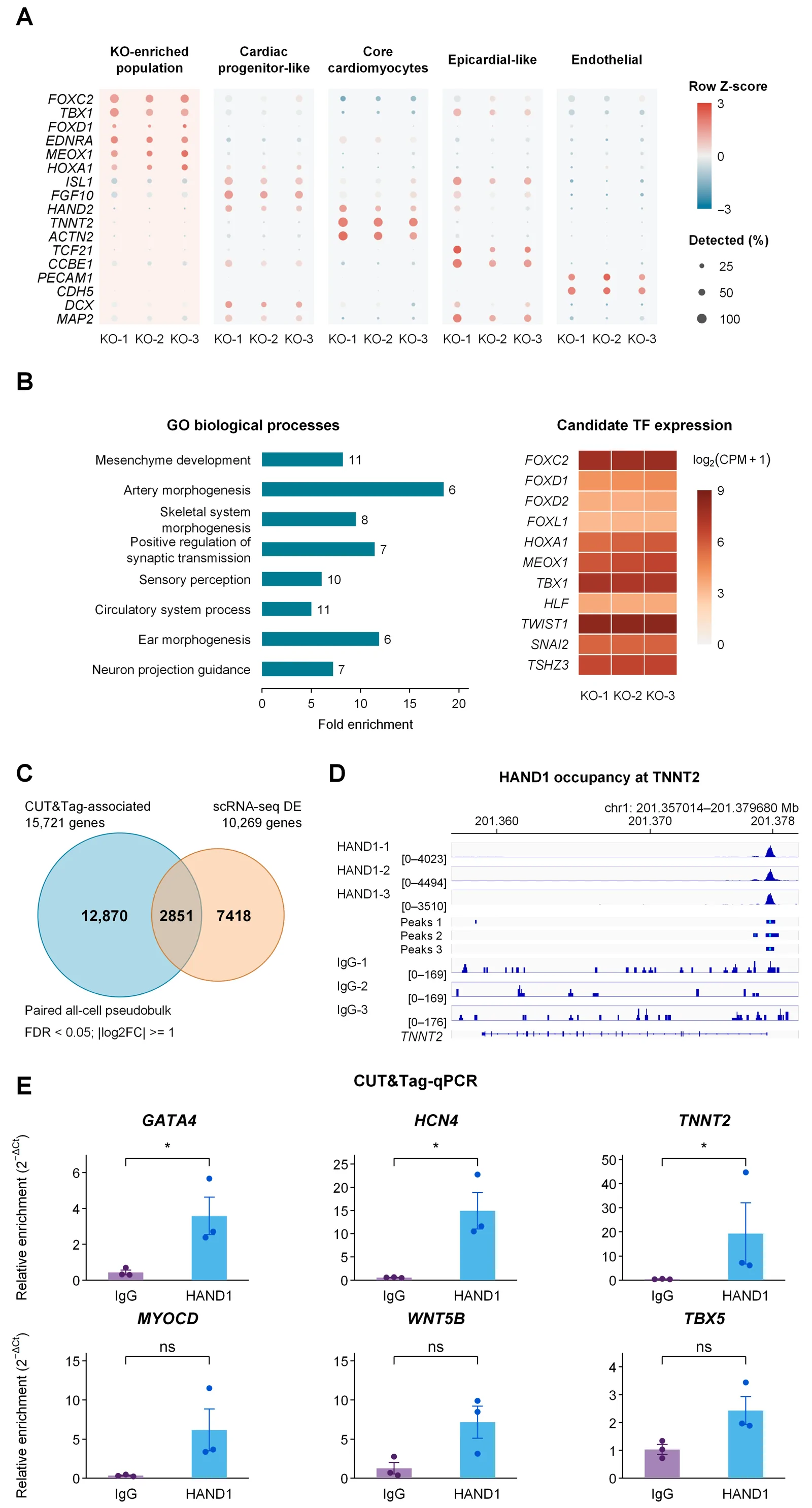
KO-enriched cell features and HAND1 chromatin occupancy. (**A**) Marker expression in the KO-enriched population and cardiac progenitor-like, core cardiomyocyte, epicardial-like, and endothelial comparator populations, shown separately for three KO samples. Color indicates the gene-wise Z-score of mean expression, and dot area indicates transcript-detection frequency. (**B**) GO biological-process enrichment of 82 consistent target-population markers (left; horizontal axis, fold enrichment; bar-end numbers, gene counts) and expression of 11 transcription-factor genes in the target population (right; log_2_[CPM + 1], without row scaling). The focused target population and its relationship to the broader compartment and exploratory transcriptional states are shown in Figure S8A. (**C**) Overlap between HAND1 CUT&Tag peak-associated genes and genes differentially expressed in paired, all-cell pseudobulk analysis of CON versus *HAND1*-KO samples (FDR < 0.05 and |log_2_ fold change| ≥ 1). Numbers indicate unique genes, including 2851 overlapping genes. This all-cell comparison does not separate cell-composition changes from within-cell-type expression changes. (**D**) *TNNT2*-associated IGV tracks showing three HAND1 CUT&Tag biological replicates, corresponding peak calls, and IgG controls; genomic coordinates and individual signal scales are indicated. (**E**) Locus-specific CUT&Tag-qPCR for regions associated with *GATA4*, *HCN4*, *TNNT2*, *MYOCD*, *WNT5B*, and *TBX5*. HAND1 and IgG signals are normalized to the negative genomic reference (NEG) and plotted as 2^−ΔCt^; statistical tests use ΔCt values. All assays shown are from day-9 organoids. * *p* < 0.05; ns, *p* ≥ 0.05. (**E**) includes three independent biological samples; technical triplicates were averaged within each sample before analysis. Error bars indicate SEM.

We characterized the expression profile of the KO-enriched population annotated as mesodermal/mesenchymal-like cells (Figure 6A). We compared this target population with the remaining cells within each KO sample and selected 82 candidate marker genes based on their expression levels and the consistency of enrichment direction across three independent differentiation batches. Functional enrichment analysis identified biological processes including mesenchyme development, artery morphogenesis, and skeletal system morphogenesis, together with terms related to neurite guidance and sensory processes. Transcription factor annotation identified 11 TF-encoding genes, including *FOXC2*, *TBX1*, *HOXA1*, *MEOX1*, *TWIST1*, and *SNAI2*; their transcript expression across the three KO samples is shown in Figure 6B. The focused KO-enriched population analyzed here is a subset of the broader mesodermal/mesenchymal-like annotation category used for cell-composition summaries (Table S6); its marker profile therefore does not necessarily represent all cells within that category. To investigate internal heterogeneity, we reanalyzed all 20,501 cells in the broader compartment, including 456 CON and 20,045 KO cells. Six provisional transcriptional states (P01–P06) were identified (Figure S8A,B). P01–P05 contained cells from all three KO samples, whereas P06 was predominantly CON-derived. Sample-resolved expression distinguished a predominantly G1-associated state (P01), S-phase- and G2/M-associated states (P02 and P03), a *RELN*/*ALDH1A2*-associated state (P04), and an extracellular-matrix-associated state with neural-related features (P05; Figure S8C). Comparison with a human Carnegie stage 7 embryo reference [34] showed modest, closely ranked similarities to several mesodermal populations (Spearman ρ = 0.299–0.398; Figure S8D). Clustering sensitivity analyses indicated variable boundary stability, particularly between P02 and P03 (Figure S8E). These findings support heterogeneous transcriptional states rather than six distinct developmental lineages.

We next examined the cellular expression patterns of six genes associated with candidate HAND1-occupied regions (Figure S5A–F). *TNNT2* and *TBX5* expression was concentrated in cardiomyocyte-associated regions of the control embedding, whereas the corresponding regions contained markedly fewer cells in the KO group. Meanwhile, *MYOCD*, *GATA4*, and *WNT5B* remained detectable in some non-cardiomyocyte regions of the KO embedding, with *WNT5B* showing a broad expression distribution within the KO-enriched region (Figure S5A–F).

Integration of paired all-cell scRNA-seq pseudobulk differential expression with genes associated with shared HAND1 CUT&Tag peaks identified 2851 overlapping genes (Figure 6C). The six loci selected for further occupancy assessment were prioritized based on cardiac developmental relevance and CUT&Tag evidence rather than this intersection alone; *WNT5B* did not meet the differential-expression fold-change threshold. Global peak overlap, genomic annotations, and signal distributions are presented in Figure S6A–D. Representative IGV tracks showed HAND1 CUT&Tag signals at selected cardiac-associated regions (Figure 6D and Figure S7A–E). Locus-specific CUT&Tag-qPCR revealed significant enrichment relative to IgG at the tested GATA4-, HCN4-, and TNNT2-associated regions, whereas differences at MYOCD-, WNT5B-, and TBX5-associated regions did not reach statistical significance (Figure 6E). Together, these analyses characterize the transcriptional heterogeneity associated with HAND1 loss and identify candidate occupied regions for further investigation of cardiac differentiation.

## 4. Discussion

A major challenge in studying early cardiac development is distinguishing changes in cardiomyocyte differentiation from broader alterations in the cellular populations that emerge during tissue formation. Human cardiac organoids offer an opportunity to examine these processes together, provided that their developmental characteristics are first established. In this study, we characterized a multicellular cardiac organoid system with stage-dependent transcriptional programs and used it to investigate developmental perturbations. The most prominent finding was that reduced TNNT2-positive tissue in the *HAND1*-knockout model was accompanied by markedly lower cardiomyocyte proportions and relative enrichment of mesodermal/mesenchymal-like cells across three paired differentiation experiments. Thus, the phenotype was not restricted to altered expression of a cardiac marker but involved a substantial difference in the cellular composition of the resulting organoids. Establishing the differentiation reference was particularly relevant given the context-dependent effects of WNT signaling [6,9,18,19,20] and variation in differentiation propensity among pluripotent stem cell lines [47]. By combining morphological, functional, and transcriptomic measurements, this model allows developmental abnormalities to be examined at both tissue and cellular levels, extending the information provided by functional readouts alone [48,49].

At 0.01 μg/mL, thalidomide exposure reduced organoid projected area and calcium transient amplitude, despite no statistically significant difference in ATP-dependent luminescence. This divergence highlights how morphological and functional alterations may be detected without a corresponding significant change in a global metabolic readout. At 10 μg/mL, transcriptomic analysis revealed broader changes in cardiac-associated gene expression, including reduced *HAND1* expression. Given the established involvement of HAND1 in early cardiac development, this observation motivated a separate genetic investigation into its contribution to organoid differentiation. Importantly, thalidomide has multiple developmental targets, including pathways involving cereblon and SALL4 [50,51,52], and the transcriptional changes detected at 10 μg/mL cannot be assumed to explain the calcium phenotype observed at 0.01 μg/mL. The pharmacological experiment therefore served two complementary purposes: demonstrating the responsiveness of the organoid system to developmental perturbation and identifying HAND1 as a candidate for further investigation, without establishing it as a mediator of thalidomide action.

Examining *HAND1* genetically revealed a differentiation phenotype that was more pronounced than might be expected from some previous human models. HAND1 has been implicated in cardiac progenitor specification and cardiomyocyte proliferation [27], yet cardiomyocyte formation has also been observed following HAND1 loss, accompanied by altered heart-field and subtype characteristics [28]. Another study reported that HAND1 perturbation could relieve BMP-dependent inhibition of cardiomyocyte differentiation under particular conditions [29]. These observations suggest that the effects of HAND1 on cardiac differentiation depend on the differentiation context. Our three-dimensional protocol combined 6 μM CHIR99021, 1.25 ng/mL BMP4, and 1 ng/mL Activin A with staged WNT modulation, creating a signaling environment that may differ from those used in other systems. Variations in cellular background, signaling conditions, tissue organization, and sampling stage could therefore contribute to the different outcomes. Nevertheless, constitutive knockout and three-dimensional culture have also been used in previous studies [28,29], making it unlikely that either feature alone explains the discrepancy. In our model, developmental gene-expression differences were already apparent by day 3, and selected cardiac-associated abnormalities remained detectable at day 15. This temporal pattern suggests that the day-9 phenotype reflects a broader disturbance of differentiation, although identifying the stage at which the divergence begins will require longitudinal analysis.

An important aspect of this disturbance was that it extended beyond the loss of cardiomyocyte representation. The relative proportions of epicardial-like and cardiac progenitor-like cells were also lower, while mesodermal/mesenchymal-like cells became the predominant population among the recovered KO cells. Because cardiac tissue formation depends on interactions among myocardial, progenitor, and non-myocardial populations, changes in these compartments may be relevant to the accompanying tissue abnormalities. Previous studies of proepicardial development and cell–matrix interactions provide a basis for examining these relationships [53,54]. At the transcriptional level, the KO-enriched cells expressed developmental regulators such as TBX1, FOXC2, and HOXA1, which have been implicated in mesodermal development and regional patterning [55,56,57,58,59]. However, these markers are not exclusive to a single cardiac lineage, and the stage-dependent expression of genes such as HCN4 further complicates direct assignment of heart-field identity [60]. Consistent with this complexity, our focused reanalysis revealed multiple transcriptional states within the broader mesodermal/mesenchymal-like compartment (Figure S8). These states included cell-cycle-associated, extracellular-matrix-related, and other developmental expression features, with several represented across independent KO samples. However, their boundaries were not equally stable and comparisons with an early human embryonic reference showed modest, closely ranked similarities to several mesodermal categories. The reference also differs in developmental stage and lacks dedicated epicardial and neuronal annotations. Taken together, these observations favor interpretation of the KO-enriched compartment as a heterogeneous collection of developmental states rather than a single expanded heart-field or outflow-tract lineage. Determining whether these states reflect altered progenitor differentiation, persistence of alternative states, or differences in cell proliferation and recovery remains an important question.

We combined expression analysis with HAND1 CUT&Tag profiling to identify candidate regulatory associations in the altered differentiation state. HAND1-associated peaks were distributed across promoter, intronic, and intergenic regions (Figure S6B), indicating that potential regulatory associations are not confined to promoter-proximal sequences. Replicate-supported occupancy signals were detected at cardiac-associated loci, including regions associated with TNNT2 and MYOCD, while locus-specific CUT&Tag-qPCR showed significant enrichment at selected GATA4-, HCN4-, and TNNT2-associated regions. In contrast, the tested MYOCD-, WNT5B-, and TBX5-associated regions did not show statistically significant qPCR enrichment. Integration with differential-expression data further identified candidate genes associated with both altered expression and HAND1-bound genomic regions, providing a starting point for prioritizing regulatory relationships. However, transcription-factor occupancy does not necessarily translate into altered transcription [61], and genomic proximity alone cannot establish direct regulatory targets. Functional perturbation of selected regions will therefore be necessary to determine whether HAND1 occupancy contributes to the observed expression and cellular phenotypes [62,63]. Nonetheless, these genomic findings complement the cellular analyses by identifying specific candidate regions through which HAND1-associated differentiation programs can be investigated.

Although the combined analyses provide a more comprehensive view of the phenotype, several questions remain unresolved. Most importantly, the genetic comparisons involved one characterized *HAND1*-knockout clone. The consistent direction of the observations across three independently performed differentiation experiments supports reproducibility within this clone pair, but independent knockout clones and *HAND1* re-expression are required to distinguish gene-specific effects from clonal variation. Moreover, constitutive knockout and single-cell profiling at day 9 do not reveal whether the altered cellular composition originates from differences in early specification, differentiation progression, proliferation, survival, or cell recovery. The proportions reported here are relative to retained cells and do not establish absolute population expansion. The small number of paired biological replicates also limits inferential precision, while the whole-sample pseudobulk comparisons remain influenced by cellular composition [64]. Additional measurement considerations include the use of separate batches and denominators for flow cytometry and scRNA-seq, the two-dimensional nature of projected TNNT2-positive area measurements, and the inability of ATP-dependent luminescence to distinguish among changes in viable cell number, tissue size, and metabolic state. Representative CON/KO calcium recordings likewise do not establish quantitative functional differences across biological replicates. Finally, the CUT&Tag-qPCR negative genomic reference requires independent validation, and the candidate regulatory associations remain to be tested functionally. Addressing these questions through independent genetic models, longitudinal profiling, quantitative functional measurements, and targeted perturbation of occupied regions will be important for determining how HAND1-associated cellular abnormalities emerge during human cardiac differentiation.

## 5. Conclusions

This study establishes a human cardiac organoid model for investigating developmental perturbations through integrated tissue-level, cellular, and transcriptional analyses. Thalidomide exposure demonstrated differentiation-stage responses and identified *HAND1* as a candidate for separate genetic investigation. In the characterized *HAND1*-knockout model, reduced cardiomyocyte-containing tissue accompanied markedly lower cardiomyocyte proportions, relative enrichment of mesodermal/mesenchymal-like cells, and selected expression abnormalities persisting to day 15. CUT&Tag and locus-specific qPCR added occupancy evidence at selected cardiac-associated regions. By placing impaired cardiomyocyte formation within its broader cellular context, the study provides a basis for investigating *HAND1*-associated differentiation abnormalities and prioritizing the associated cellular states and occupied regions for further functional study.

## Data Availability

The raw sequence data from human iPSC-derived cardiac differentiation cultures reported in this paper have been deposited in the Genome Sequence Archive [65] in National Genomics Data Center [66], China National Center for Bioinformation/Beijing Institute of Genomics, Chinese Academy of Sciences (GSA-Human: HRA019739); the data are accessible at https://ngdc.cncb.ac.cn/gsa-human/browse/HRA019739 (accessed on 20 October 2026). The raw data are available upon reasonable request through the GSA-Human Data Access Committee (DAC), for which the corresponding author serves as the contact. Shared URL: https://ngdc.cncb.ac.cn/gsa-human/s/tXbgf7FM (accessed on 20 October 2026). Processed HAND1 CUT&Tag peak calls, CUT&Tag-qPCR primer-design information, and single-cell annotation and summary tables (cell composition, sample-by-cluster expression, quality control, cell-cycle state, and gene co-detection) are available in Zenodo (https://doi.org/10.5281/zenodo.23439175; accessed on 20 October 2026).

## Supplementary Materials

The following supporting information can be downloaded at: https://www.mdpi.com/article/10.3390/cells15211948/s1, Figure S1: Flow-cytometry gating and transcriptional dynamics during cardiac organoid differentiation; Figure S2: Additional thalidomide-response data, *HAND1*-knockout validation, and single-cell RNA-sequencing quality control; Figure S3: *HAND1* expression dynamics, developmental gene expression, and cell composition in control and *HAND1*-KO organoids; Figure S4: Representative fluorescence recordings from control and *HAND1*-KO cardiac organoids; Figure S5: Single-cell expression distributions of genes associated with candidate HAND1-occupied regions; Figure S6: HAND1 CUT&Tag peak overlap, genomic annotations, and signal distributions; Figure S7: HAND1 CUT&Tag occupancy at additional cardiac-associated genomic regions; Figure S8: Transcriptional heterogeneity within the mesodermal/mesenchymal-like compartment and exploratory reference comparisons; Table S1: Antibodies used in this study; Table S2: Primers used for RT-qPCR; Table S3: Primers used for Sanger sequencing; Table S4: Selected HAND1 CUT&Tag peaks, primers, and amplicon mapping; Table S5: Per-sample cell retention and quality metrics; Table S6: Sample-level cell composition and paired changes; Video S1: Representative calcium transient activity in control and thalidomide-treated cardiac organoids.

## Abbreviations

The following abbreviations are used in this manuscript:

CUT&Tag	cleavage under targets and tagmentation
DMSO	dimethyl sulfoxide
HAND1	heart and neural crest derivatives expressed 1
hiPSCs	human induced pluripotent stem cells
IgG	immunoglobulin G
KO	knockout
PCA	principal component analysis
UMAP	uniform manifold approximation and projection
