# Supplementary material for: Human Cardiac Organoids Reveal HAND1 Loss-Associated Alterations in Cell Composition During Early Cardiac Differentiation
Source: Cells. 2026 Oct 26;15(21):1948. doi: 10.3390/cells15211948 (PMC13761964; doi:10.3390/cells15211948)
Supplement: Supplementary file 1 [file cells-15-01948-s001.zip › Supplementary_Figures.pdf]

Figure S1

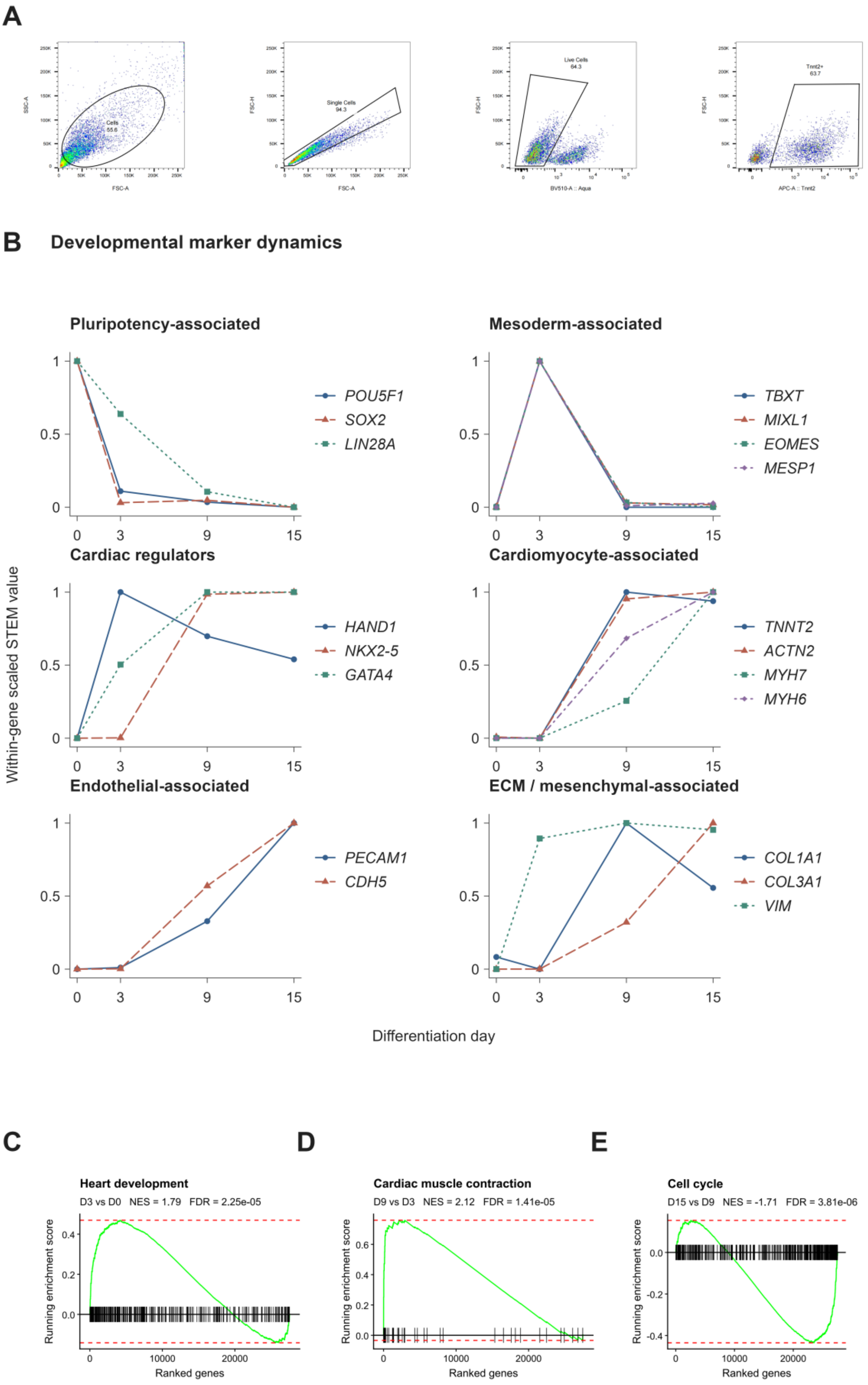
A
Cells 55.6
SSC-A
FSC-A
Single Cells 94.3
FSC-H
FSC-A
Live Cells 64.3
FSC-H
BV510-A :: Aqua
Tnnt2+ 63.7
FSC-H
APC-A :: Tnnt2
B
Developmental marker dynamics
Pluripotency-associated
POU5F1
SOX2
LIN28A
Mesoderm-associated
TBXT
MIXL1
EOMES
MESP1
Cardiac regulators
HAND1
NKX2-5
GATA4
Cardiomyocyte-associated
TNNT2
ACTN2
MYH7
MYH6
Endothelial-associated
PECAM1
CDH5
ECM / mesenchymal-associated
COL1A1
COL3A1
VIM
Within-gene scaled STEM value
Differentiation day
C
Heart development
D3 vs D0 NES = 1.79 FDR = 2.25e-05
Running enrichment score
Ranked genes
D
Cardiac muscle contraction
D9 vs D3 NES = 2.12 FDR = 1.41e-05
Running enrichment score
Ranked genes
E
Cell cycle
D15 vs D9 NES = -1.71 FDR = 3.81e-06
Running enrichment score
Ranked genes

Figure S1. Flow-cytometry gating and transcriptional dynamics during cardiac organoid differentiation.

(A) Representative gating sequence for the day-9 CON sample showing 63.7% TNNT2-positive viable singlets (Figure 2C). Cells were sequentially selected by FSC-A/SSC-A, FSC-A/FSC-H, and Aqua-low fluorescence (BV510-A), followed by TNNT2 detection in APC-A. Gate percentages are relative to the immediate parent population. **(B)** Descriptive expression trajectories of 19 genes across differentiation days 0, 3, 9, and 15. STEM-derived values were independently rescaled to 0–1 within each gene, illustrating relative temporal patterns rather than comparable expression levels between genes. **(C–E)** Representative GSEA curves for heart development (D3 versus D0), cardiac muscle contraction (D9 versus D3), and cell cycle (D15 versus D9), respectively. Genes were ranked by DESeq2 Wald statistics from three independent differentiation samples per time point. Black ticks indicate gene-set members; normalized enrichment scores (NES) and false discovery rates (FDR) are shown. Positive and negative NES indicate enrichment toward higher expression at the later and earlier stages, respectively.

Figure S2

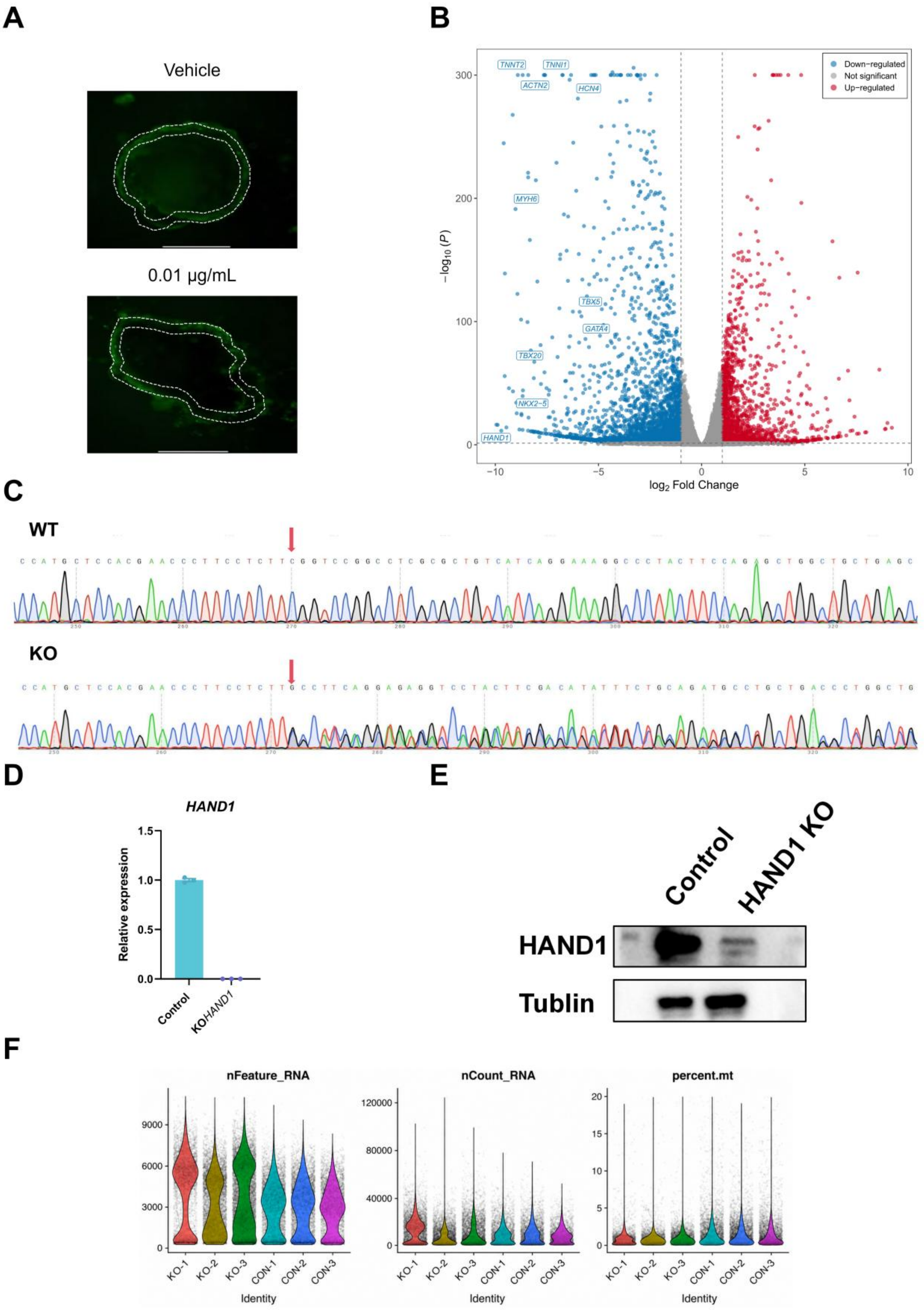
A
Vehicle
0.01 μg/mL
B
Down-regulated
Not significant
Up-regulated
TNNT2
TNNI1
ACTN2
HCN4
MYH6
TBX5
GATA4
TBX20
NKX2-5
HAND1
−log10(P)
log2 Fold Change
C
WT
KO
D
HAND1
Relative expression
Control
KOHAND1
E
Control
HAND1 KO
HAND1
Tublin
F
nFeature_RNA
nCount_RNA
percent.mt
KO-1
KO-2
KO-3
CON-1
CON-2
CON-3
Identity

Figure S2. Additional thalidomide-response data, *HAND1*-knockout validation, and single-cell RNA-sequencing quality control.

(A) Representative Fluo-4 fluorescence images from day-9 cardiac organoids exposed to vehicle or 0.01 μg/mL thalidomide, with dashed outlines marking the regions used for calcium-signal analysis. **(B)** Volcano plot of bulk RNA-seq differential expression in day-9 organoids exposed to 10 μg/mL thalidomide versus vehicle (three independent biological replicates per group). Differential expression was analyzed using DESeq2 with $|\log_2$ fold change$| \geq 1$ and FDR < 0.05. Red and blue indicate upregulated and downregulated genes, respectively; gray indicates genes not meeting these criteria. The vertical axis displays $-\log_{10}$ of the nominal *p* value, whereas the color classification uses FDR-adjusted *p* values. Selected cardiac-associated genes are labeled. **(C)** Sanger sequencing chromatograms of the *HAND1* target region in wild-type (WT) and *HAND1*-KO cells; red arrows mark the corresponding target-region positions. **(D)** Relative *HAND1* mRNA expression measured by RT-qPCR, normalized to *GAPDH* and the control group (control = 1). Points represent independent biological replicates ($n = 3$); data are mean ± SEM. **(E)** Representative Western blot of HAND1 protein, with tubulin as the loading control. **(F)** Distributions of detected gene numbers (nFeature_RNA), total UMI counts (nCount_RNA), and mitochondrial transcript percentages (percent.mt) in six day-9 single-cell samples. Points represent individual cells. CON-1–3 and KO-1–3 originate from three independent differentiation experiments; matching numbers identify paired samples.

Figure S3

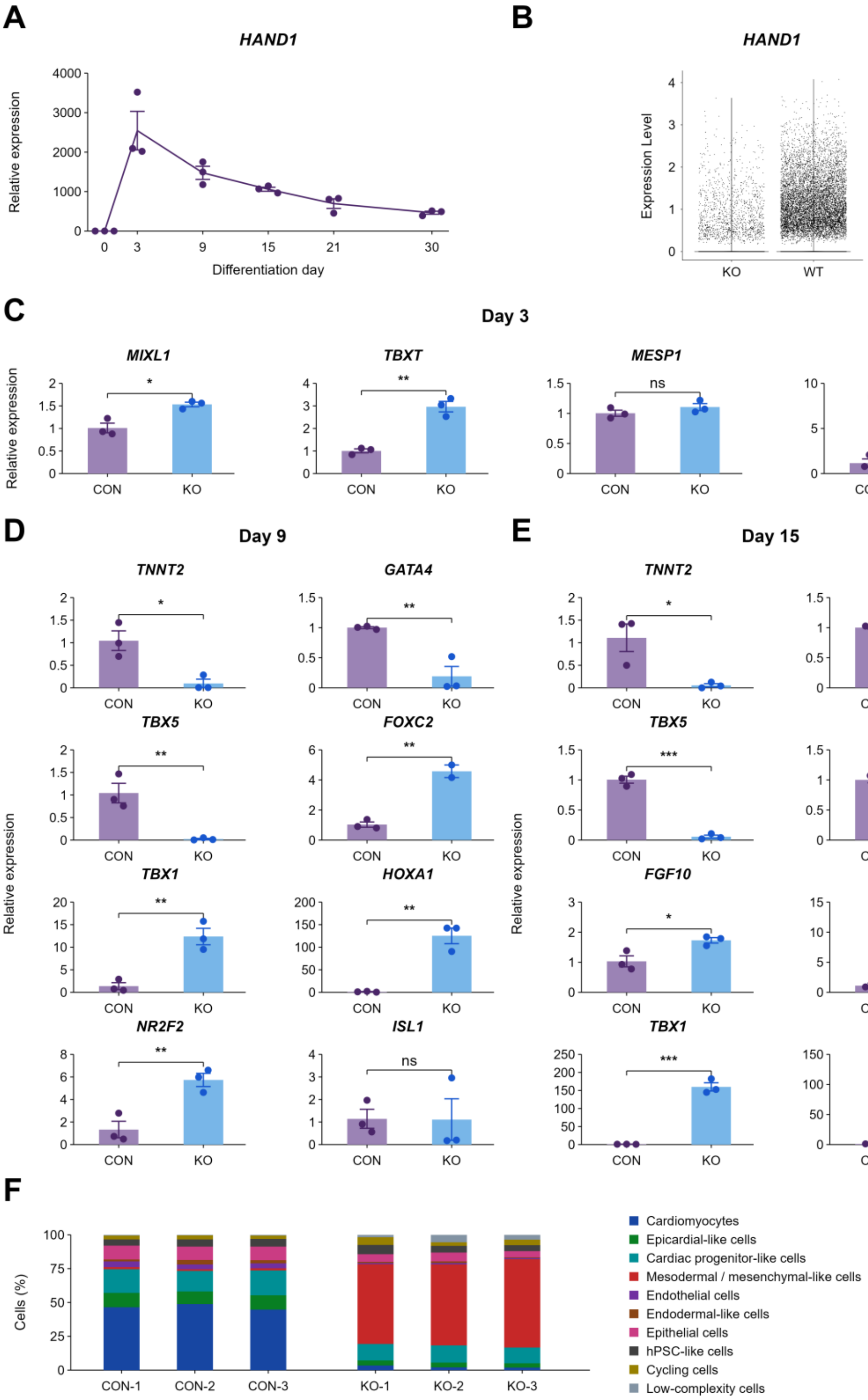
A
HAND1
Relative expression
Differentiation day
B
HAND1
Expression Level
KO
WT
C
Day 3
MIXL1
TBXT
MESP1
HOXB1
CON
KO
ns
D
Day 9
TNNT2
GATA4
TBX5
FOXC2
TBX1
HOXA1
NR2F2
ISL1
E
Day 15
NKX2-5
MYL7
FGF10
FGF8
F
Cells (%)
CON-1
CON-2
CON-3
KO-1
KO-2
KO-3
Cardiomyocytes
Epicardial-like cells
Cardiac progenitor-like cells
Mesodermal / mesenchymal-like cells
Endothelial cells
Endodermal-like cells
Epithelial cells
hPSC-like cells
Cycling cells
Low-complexity cells

Figure S3. ***HAND1*** **expression dynamics, developmental gene expression, and cell composition in control and** ***HAND1*****-KO organoids.**

(A) RT-qPCR analysis of *HAND1* expression in control organoids on differentiation days 0, 3, 9, 15, 21, and 30. **(B)** Distribution of *HAND1* transcript expression in day-9 control and *HAND1*-KO cells from the single-cell RNA-seq dataset; WT denotes the control group (CON), and points represent cells rather than independent biological replicates. **(C)** Day-3 RT-qPCR analysis of *MIXL1*, *TBXT*, *MESP1*, and *HOXB1*. **(D)** Day-9 RT-qPCR analysis of *TNNT2*, *GATA4*, *TBX5*, *FOXC2*, *TBX1*, *HOXA1*, *NR2F2*, and *ISL1*. **(E)** Day-15 RT-qPCR analysis of *TNNT2*, *NKX2-5*, *TBX5*, *MYL7*, *FGF10*, *NR2F2*, *TBX1*, and *FGF8*. RT-qPCR values were normalized to *GAPDH*, with day 0 as the calibrator in A and the corresponding CON group as the calibrator in C–E. Each point represents one independent differentiation experiment. Data are mean ± SEM of three experiments, except for *FOXC2* in the day-9 KO group, which has two valid biological replicates. Statistical analyses are described in the Methods; brackets indicate the comparisons shown. *$p < 0.05$, **$p < 0.01$, ***$p < 0.001$, ****$p < 0.0001$; ns, $p \geq 0.05$. **(F)** Cell-composition profiles of all six day-9 samples, including 63,954 retained cells in total. Each bar represents one sample, and each category is expressed as a percentage of all retained cells in that sample. Matching CON and KO sample numbers denote paired differentiation batches.

Figure S4

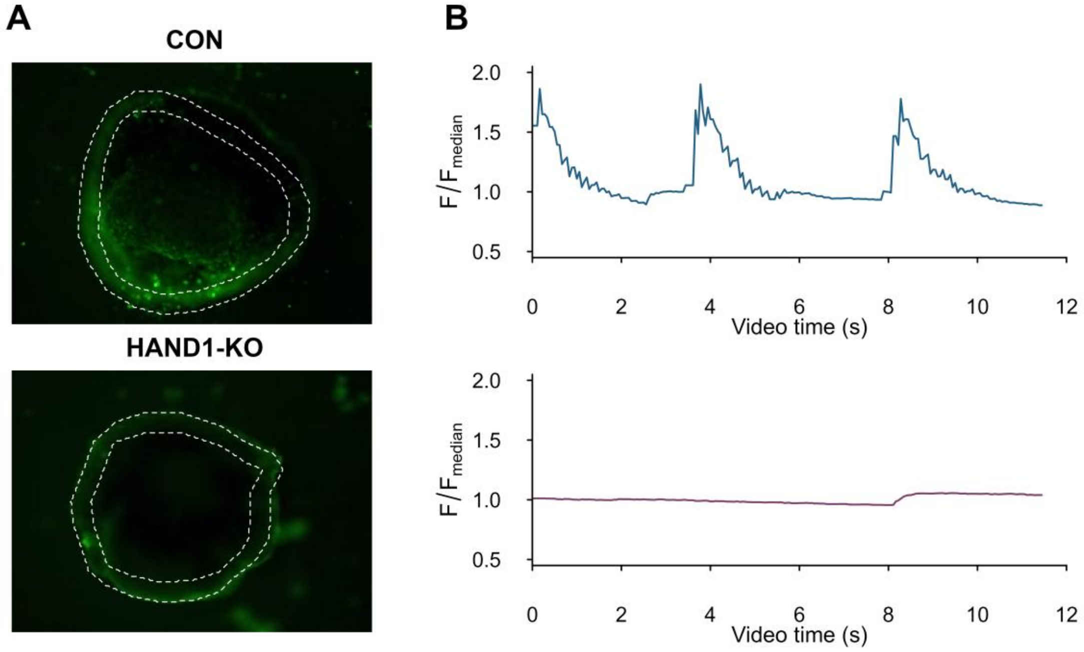

Figure S4. Representative fluorescence recordings from control and HAND1-KO cardiac organoids.

(A) Representative Fluo-4 fluorescence images of untreated day-9 CON and *HAND1*-KO organoids. Dashed outlines indicate fixed peripheral tissue ROIs. **(B)** Corresponding fluorescence traces from one matched CON/KO pair, representative of recordings obtained in three independent differentiation experiments. Both recordings were acquired using the same dye and imaging settings within the batch. The first 207 frames are displayed with identical axis limits. Mean ROI fluorescence was normalized to the median fluorescence over the displayed interval (F/F_{median}), without background subtraction, smoothing, or detrending. Time was calculated from the encoded AVI frame rate (18 frames/s; approximately 11.5 s). The KO baseline shift coincided with background variation and was not interpreted as a calcium transient. No pooled amplitude or frequency comparison was performed.

Figure S5

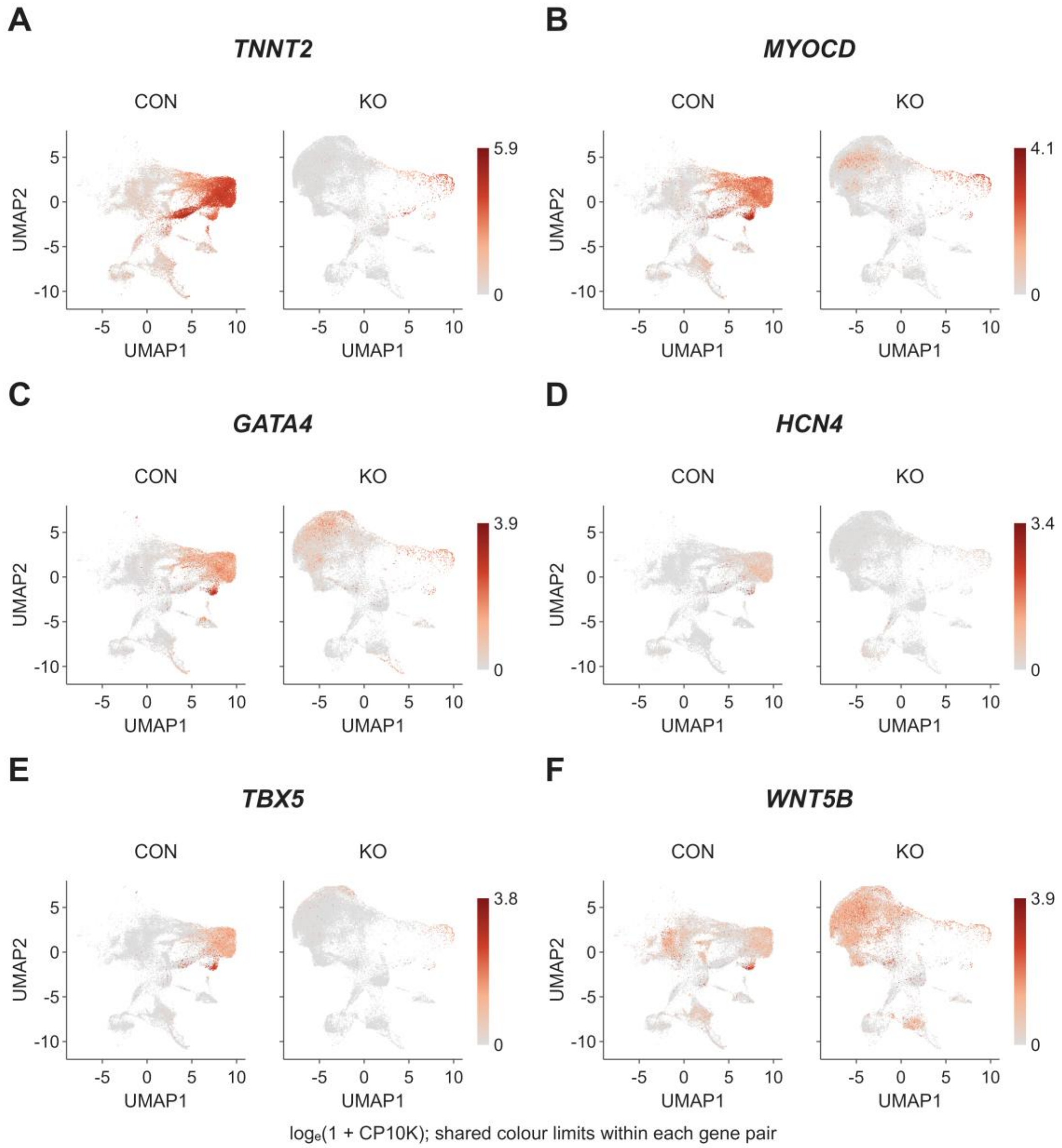

Figure S5. Single-cell expression distributions of genes associated with candidate HAND1-occupied regions.

(A–F) Expression of *TNNT2* **(A)**, *MYOCD* **(B)**, *GATA4* **(C)**, *HCN4* **(D)**, *TBX5* **(E)**, and *WNT5B* **(F)** in day-9 CON and *HAND1*-KO organoids. All retained cells are displayed in a shared UMAP embedding, split by condition. Colors indicate log$_e$(1 + CP10K), where CP10K represents transcript counts normalized to 10,000 per cell. Each gene uses a common color scale and coordinate limits across conditions. Data comprise three paired differentiation experiments per condition. These plots describe cellular expression distributions without distinguishing cell-composition effects from within-cell-type expression changes.

Figure S6

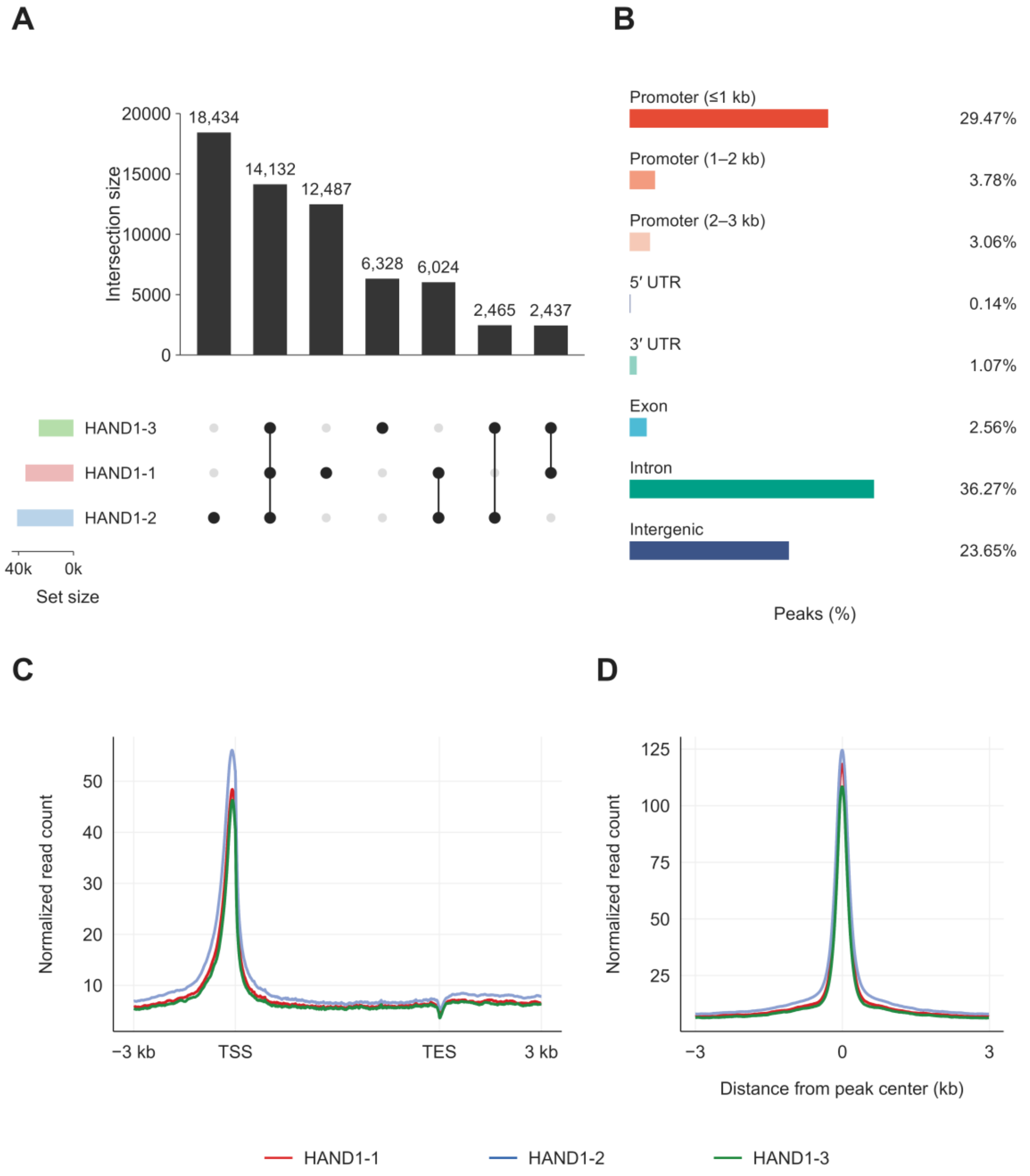

Figure S6. HAND1 CUT&Tag peak overlap, genomic annotations, and signal distributions.

(A) UpSet plot of HAND1 CUT&Tag peak sets from three biological replicates of day-9 cardiac organoids. Horizontal bars indicate the size of each replicate's peak set, and upper bars indicate the intersection sizes for the replicate combinations marked by the dot matrix. Counts refer to genomic peak regions, not genes. **(B)** Genomic annotation of HAND1 CUT&Tag peaks, including promoter regions at the indicated distances, 5′ and 3′ untranslated regions (UTRs), exons, introns, and intergenic regions. Percentages indicate the proportion of analyzed peaks assigned to each category. **(C)** Normalized CUT&Tag signal across gene bodies and the 3-kb regions upstream of transcription start sites (TSS) and downstream of transcription end sites (TES). **(D)** Normalized signal within 3 kb on either side of peak centers. Line colors in C and D identify HAND1-1, HAND1-2, and HAND1-3.

Figure S7

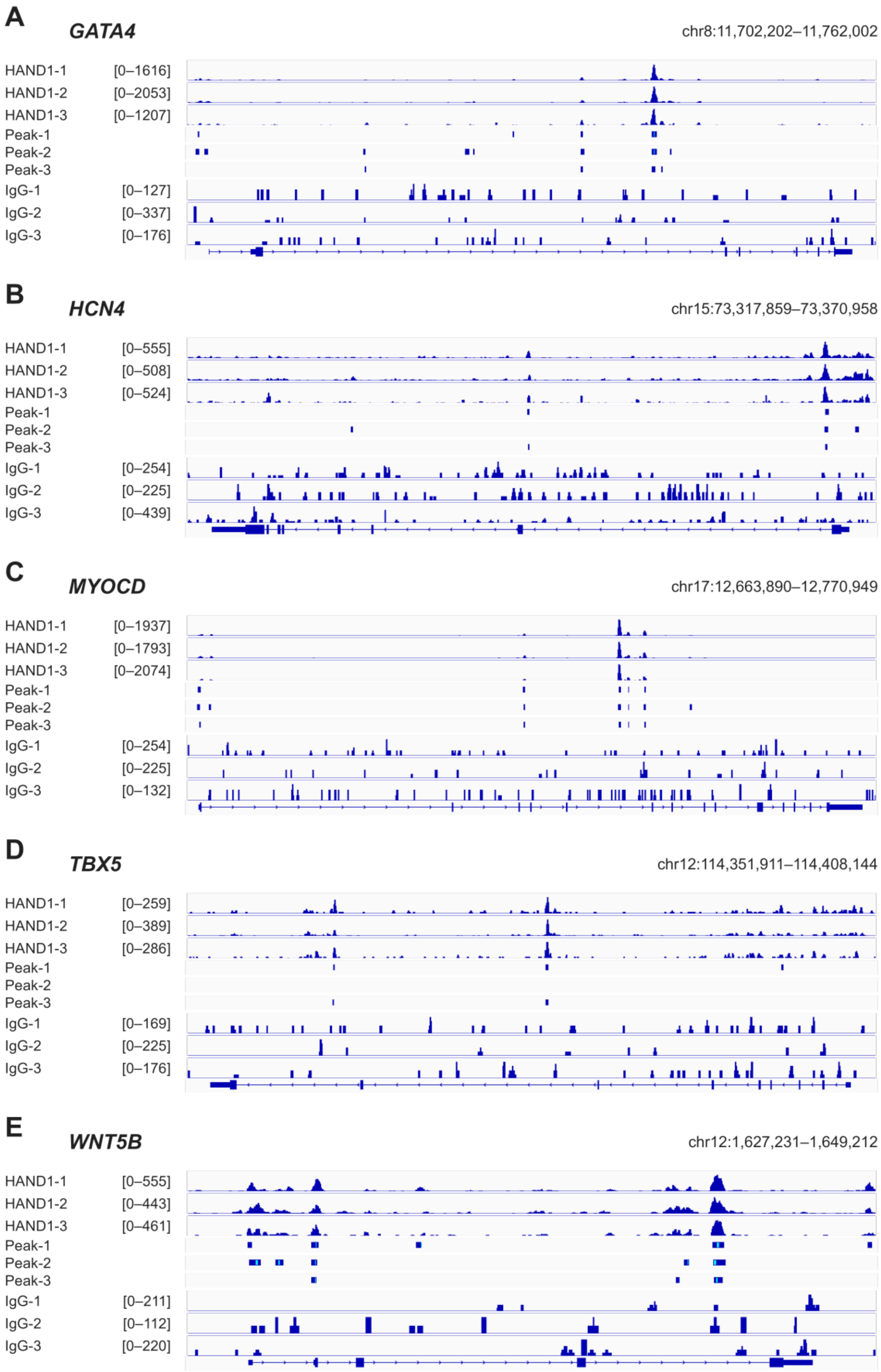

Track scales shown as [minimum–maximum]; original individual scales retained

Figure S7. HAND1 CUT&Tag signals at additional cardiac-associated genomic regions.

(A–E) Representative Integrative Genomics Viewer (IGV) tracks at regions associated with *GATA4* **(A)**, *HCN4* **(B)**, *MYOCD* **(C)**, *TBX5* **(D)**, and *WNT5B* **(E)** in day-9 cardiac organoids. Each panel displays HAND1 CUT&Tag signals from three biological replicates, corresponding peak calls, IgG controls, and gene annotations. Genomic coordinates and individual signal scales are indicated. Tracks use independent intensity scales; peak heights should not be compared directly across tracks.

Supplementary Methods for CUT&Tag data analysis

Raw reads were filtered to remove adapter-containing, poly-N, and low-quality reads. Quality-filtered reads were aligned using BWA, and uniquely mapped reads were used for MACS2 peak calling ($q < 0.05$). BEDTools assigned genomic features by ≥1-bp overlap, prioritizing promoter bins within 1 kb, 1–2 kb, and 2–3 kb of the TSS on either side, followed by 5′ UTR, 3′ UTR, exon, intron, and intergenic regions. In the supplied shared-peak annotation table, peak centers were associated with the nearest Ensembl transcript TSS within 100 kb; tied assignments were split and gene symbols deduplicated. Exact gene-symbol matching identified the overlap with scRNA-seq pseudobulk differentially expressed genes. Raw UMI counts from all retained cells were summed per biological sample and analyzed using DESeq2 (~ pair + group), with FDR < 0.05 and $|\log_2 \text{fold change}| \geq 1$. This overlap is descriptive, not an enrichment test, and retains cell-composition effects.

Figure S8

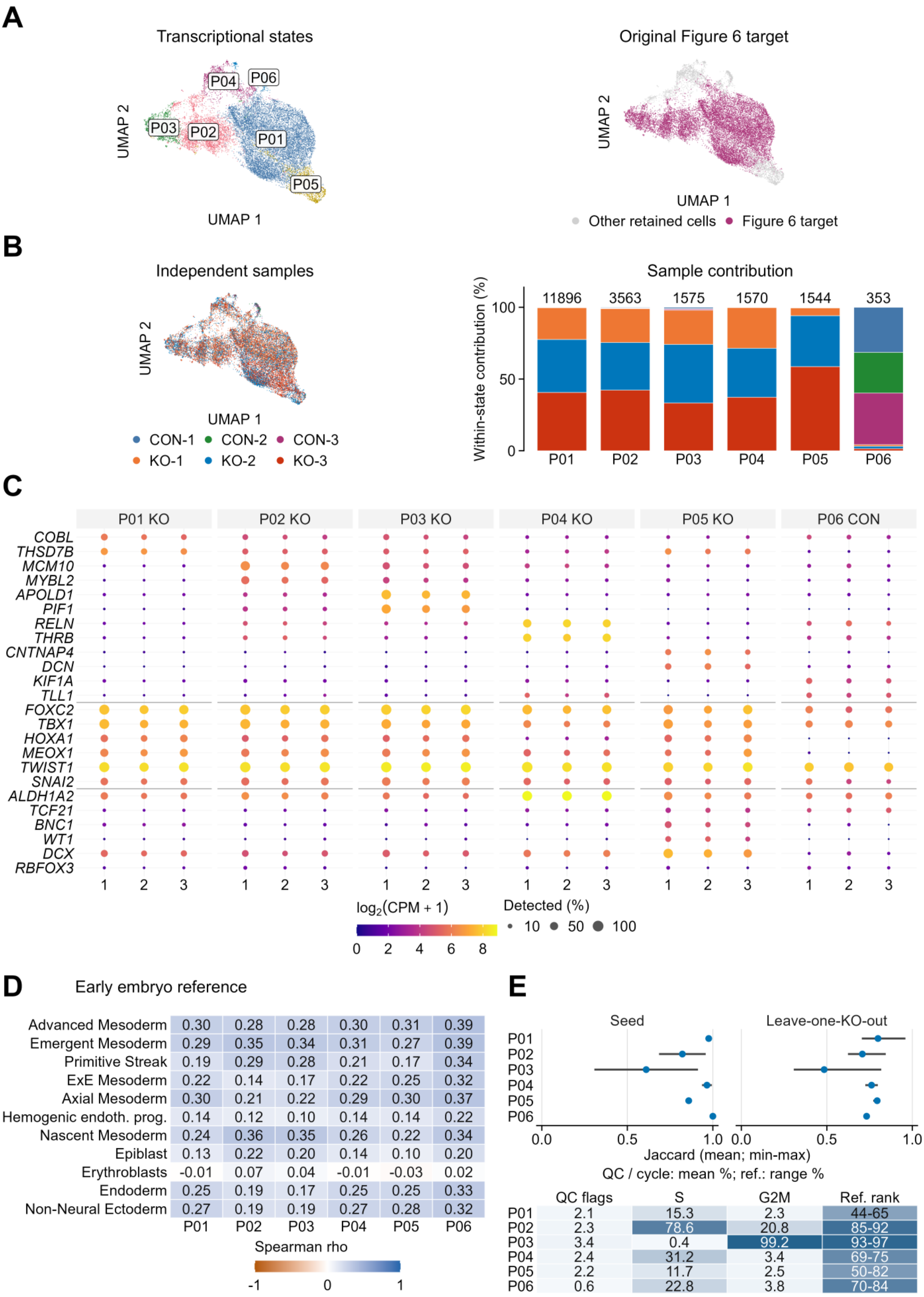
A
Transcriptional states
P04
P06
P03
P02
P01
P05
UMAP 2
UMAP 1
Original Figure 6 target
UMAP 2
UMAP 1
Other retained cells
Figure 6 target
B
Independent samples
UMAP 2
UMAP 1
CON-1
CON-2
CON-3
KO-1
KO-2
KO-3
Sample contribution
Within-state contribution (%)
11896
3563
1575
1570
1544
353
100
50
0
P01
P02
P03
P04
P05
P06
C
P01 KO
P02 KO
P03 KO
P04 KO
P05 KO
P06 CON
COBL
THSD7B
MCM10
MYBL2
APOLD1
PIF1
RELN
THRB
CNTNAP4
DCN
KIF1A
TLL1
FOXC2
TBX1
HOXA1
MEOX1
TWIST1
SNAI2
ALDH1A2
TCF21
BNC1
WT1
DCX
RBFOX3
1 2 3
log2(CPM + 1)
0 2 4 6 8
Detected (%)
10 50 100
D
Early embryo reference
Advanced Mesoderm 0.30 0.28 0.28 0.30 0.31 0.39
Emergent Mesoderm 0.29 0.35 0.34 0.31 0.27 0.39
Primitive Streak 0.19 0.29 0.28 0.21 0.17 0.34
ExE Mesoderm 0.22 0.14 0.17 0.22 0.25 0.32
Axial Mesoderm 0.30 0.21 0.22 0.29 0.30 0.37
Hemogenic endoth. prog. 0.14 0.12 0.10 0.14 0.14 0.22
Nascent Mesoderm 0.24 0.36 0.35 0.26 0.22 0.34
Epiblast 0.13 0.22 0.20 0.14 0.10 0.20
Erythroblasts -0.01 0.07 0.04 -0.01 -0.03 0.02
Endoderm 0.25 0.19 0.17 0.25 0.25 0.33
Non-Neural Ectoderm 0.27 0.19 0.19 0.27 0.28 0.32
P01 P02 P03 P04 P05 P06
Spearman rho
-1 0 1
E
Seed
Leave-one-KO-out
P01
P02
P03
P04
P05
P06
0.0 0.5 1.0 0.0 0.5 1.0
Jaccard (mean; min-max)
QC / cycle: mean %; ref.: range %
QC flags S G2M Ref. rank
P01 2.1 15.3 2.3 44-65
P02 2.3 78.6 20.8 85-92
P03 3.4 0.4 99.2 93-97
P04 2.4 31.2 3.4 69-75
P05 2.2 11.7 2.5 50-82
P06 0.6 22.8 3.8 70-84

Figure S8. Transcriptional heterogeneity within the mesodermal/mesenchymal-like compartment and exploratory reference comparisons.

(A) UMAP of 20,501 cells from original clusters 1, 3, 4, 15, 17, and 24 (456 CON and 20,045 *HAND1*-KO cells), colored by six provisional transcriptional states (P01–P06) identified using uncorrected principal components (left). The original Figure 6A–B target population, comprising 16,881 KO cells from clusters 1, 3, and 4, is highlighted in the same embedding (right).

(B) Sample-colored UMAP (left) and within-state sample contributions (right). Bars show the percentage contributed by each sample, with total state-cell counts indicated above. Samples represent three paired differentiation batches.

(C) Sample-resolved expression of representative state markers, six candidate transcription-factor genes, and selected developmental markers. Color indicates $\log_2$(CPM + 1) from sample–state pooled counts normalized using the full retained RNA library; dot size indicates transcript-detection frequency. P01–P05 show three KO samples, whereas P06 shows three CON samples because its KO pools contained only 4, 6, and 5 cells. *CNTNAP4* and *DCN* illustrate neural-related and extracellular-matrix-related expression in P05, respectively.

(D) Spearman correlations between sample–state expression profiles (≥20 cells per pool) and 11 annotated categories from the Tyser et al. (2021) human Carnegie stage 7 embryo reference. Correlations were calculated using 2,000 reference-selected variable genes; tiles show equal-weight means across three evaluable samples on a fixed −1 to 1 scale. These values indicate transcriptomic similarity rather than lineage identity.

(E) Clustering stability and cell-state quality summaries. Upper panels show best-match Jaccard indices across alternative random seeds (29 and 47) and leave-one-KO-sample-out analyses, relative to the displayed seed-11 states. Lower panels show sample-averaged percentages of QC-flagged, S-phase, and G2/M-phase cells. "Ref. rank" indicates the frequency with which the top reference category was retained across 100 random subsets of 80% of the reference-selected genes, rather than a classification probability. QC flags were evaluated for sensitivity analysis without removing cells from A or B. Analyses are descriptive and preserve biological samples as the units of replication.

Supplementary Methods for Figure S8

All 20,501 retained cells from original clusters 1, 3, 4, 15, 17, and 24 (456 CON; 20,045 HAND1-KO) were reanalyzed without condition balancing using R v4.5.3 and Seurat v5.4.0. The original Figure 6A–B target comprised 16,881 KO cells from clusters 1, 3, and 4. Counts were log-normalized using full-library totals and a scale factor of 10,000. VST features were selected anew (2,000 genes detected in ≥10 cells) and scaled for PCA. Neighbors used the first 30 PCs, k = 20, and SNN pruning of 1/15. Leiden clustering evaluated resolutions 0.2, 0.4, 0.6, and 0.8 with seeds 11, 29, and 47. Resolution 0.2 was the lowest multicluster setting meeting median adjusted Rand index ≥0.90 and median bidirectional best-match Jaccard index ≥0.75. Seed-11 states were visualized using UMAP (30 neighbors; min_dist = 0.3). Sensitivity checks included paired-batch Harmony v2.0.5 and leave-one-KO-sample-out reanalysis. Sample-specific three-scaled-MAD thresholds flagged low counts, low gene numbers, or high mitochondrial percentages; counts and gene numbers were $\log_{10}(1 + x)$-transformed. QC exclusions were assessed within fixed states without changing displayed cell counts or Table S6 proportions.

Descriptive markers compared each state with the remaining six-cluster cells within each sample, requiring ≥20 cells in both groups. Candidates required target CPM ≥5, detection ≥10%, and $\log_2$[(target CPM + 1)/(rest CPM + 1)] ≥1 in at least two KO samples, with no evaluable KO comparison below −0.5. P06 used analogous CON summaries because its KO pools were insufficient. Reference comparisons used the Tyser et al. (2021) human CS7 dataset (1,195 cells from one embryo; 11 annotated categories). Eligible shared genes were detected in ≥5 reference cells and ≥10% of one reference category; mitochondrial genes, numbered RPL/RPS genes, and HAND1 were excluded. The 2,000 highest-variance reference genes were selected independently of query markers. Reference centroids averaged per-cell $\log_2$(CPM + 1); query profiles pooled counts by sample and state, with full-library normalization. Pools containing ≥20 cells were compared by Spearman correlation, and equal-weight sample means were displayed. Ranking sensitivity used 100 random subsets retaining 80% of features and exclusion of 52 canonical cell-cycle genes. These analyses describe expression similarity rather than validated lineage identity.
