# Supplementary material for: Human Cardiac Organoids Reveal HAND1 Loss-Associated Alterations in Cell Composition During Early Cardiac Differentiation
Source: Cells. 2026 Oct 26;15(21):1948. doi: 10.3390/cells15211948 (PMC13761964; doi:10.3390/cells15211948)
Supplement: Supplementary file 1 [file cells-15-01948-s001.zip › Supplementary_Tables_S1-S6.pdf]

Supplementary Table S1. Antibodies used for immunofluorescence staining and western blotting

Name	Catalog No.	Dilution
Recombinant Anti-Cardiac Troponin T antibody [EPR3695]	ab91605	1:200
Goat Anti-Rabbit IgG H&L (Alexa Fluor® 555)	ab150078	1:1000
Anti-HAND1 antibody, polyclonal	10882-1-AP	1:500
Alpha Tubulin Monoclonal antibody	66031-1-Ig	1:20000
HRP-conjugated Goat Anti-Mouse IgG(H+L)	SA00001-1	1:1000
Goat Anti-Rabbit IgG (H+L) HRP	S0001	1:3000
Alexa Fluor® 647 anti-Cardiac Troponin T (EPR20266)	ab314677	1:500
Anti-HAND1, mouse monoclonal	ab279390	1:50
Goat anti-mouse IgG H&L	N270	1:100

Supplementary Table S2. Primers used for RT-qPCR and genotyping

Gene	Forward 5'-3'	Reverse 5'-3'
TBXT (*BRY*)	TGTCCCAGGTGGCTTACAGATGAA	GGTGTGCCAAAGTTGCCAATACAC
EOMES	CCCACTACAATGTGTTCGTAGA	TGCATGTTATTGTCGGCTTTG
FGF10	TGTGGAAATGGATACTGACACA	GACGGAAGACACCAAGAACA
FGF8	CTGCGTCTTCACGGAGATT	GGTGAAGGCCATGTACCAG
FOXC2	CAACATGTTCGAGAACGGCAGCTT	CGCTCTTGATCACCACCTTCTTCT
GAPDH	GACAGTCAGCCGCATCTTCT	GCGCCCAATACGACCAAATC
GATA4	CGAATGACGGCATCTGTTTGCCAT	ATTTGGTATTAGGGATGCAGGGCG
HAND1	GAACCCTTCCTCTTCGGTCC	GACCATAGGCGGTGGCG
HOXA1	GAAAGTTGGAGAGTACGGCTAC	TGTTGAAGTGGAACTCCTTCTC
HOXB1	AGAAGGAGACGGAGGCTATT	GGTCTGCTCGTTCCCATAAG
ISL1	GAAGGTGGAGCTGCATTGGTTTGA	TAAACCAGCTACAGGACAGGCCAA
MESP1	AGCCCAAGTGACAAGGGACAACT	AAGGAACCACTTCGAAGGTGCTGA
MIXL1	CTGTGCTCCTGGAACTGAAA	TGGGAGCTAGAGTCAGAGATG
MYL7	AAAGAGCGTGAGGAAGACGG	TGTATCGACCAAAACCGTGA
NKX2-5	AAGTGTGCGTCTGCCTTTCCCG	TTGTCCGCCTCTGTCTTCTCCA
NR2F2	TGATGTAGCCCATGTGGAAAG	GCTGCCGGACAGTAACATATC
TBX1	GTGAAGAAGAACGCGAAGGT	TGGTGACGATCATCTCGGT
TBX5	ACAAAGTGAAGGTGACGGGCCTTA	ATCTGTGATCGTCGGCAGGTACAA
TNNT2	AAGAGGCAGACTGAGCGGGAAA	AGATGCTCTGCCACAGCTCCTT

Supplementary Table S3. Primers used for predicted off-target validation of *HAND1* sgRNA A1

sgRNA	Predicted off-target site	Forward primer name	Forward primer sequence (5′–3′)	Reverse primer name	Reverse primer sequence (5′–3′)	Amplicon size
A1	G1	G1-F1	GCCCAATTGAGAAATAGTACCTTGG	G1-R1	GATAACACATGGGAGTGACTTTGG	590 bp
A1	G2	G2-F1	TGGCCTCAGTTATCTCGTTTGTAA	G2-R1	TCGGGAACATTCTTTTGAAATGGG	1593 bp
A1	G3	G3-F1	GAGGGTTGCAAGGATGTAATGAGA	G3-R1	ACAAGAAGAGTCTGCTACAAGCTA	523 bp
A1	G4	G4-F1	AAGTGAGGAATTCTAGCCCTGAAG	G4-R1	CTCCACGATAACCGACTTTGTCAC	402 bp
A1	G5	G5-F1	CGAGTTCGTAGATCCCAAAACAAG	G5-R1	TCTGGGCAGGATCAAAGGTCTA	919 bp

Note: The predicted off-target sites were amplified by PCR and validated by Sanger sequencing. The *HAND1* sgRNA A1 target sequence was AGCGCGAGGCCGGACCGAAG with AGG as the PAM sequence. The annealing temperature for all primer pairs was 61.0°C.

Supplementary Table S4. Parent HAND1 CUT&Tag peaks and locus-specific qPCR amplicons. Peak coordinates are the MACS2 narrowPeak calls that overlap the corresponding amplicon in each replicate. Coordinates are GRCh38/hg38, 1-based and inclusive.

Table S4a. Parent peaks by biological replicate

Locus	Tested peak class	Hand1-1 peak	Hand1-2 peak	Hand1-3 peak	Replicate support
MYOCD	Promoter/TSS-proximal	chr17:12,665,851–12,666,251	chr17:12,665,696–12,666,205	not overlapping (nearest peak 65 bp distal)	3/3 in region
TNNT2	Promoter-proximal	chr1:201,377,587–201,378,180	chr1:201,377,553–201,378,427	chr1:201,377,571–201,378,127	3/3
WNT5B	Promoter-proximal (alternative TSS, ENST00000542408)	chr12:1,631,247–1,631,501	chr12:1,631,247–1,631,454	chr12:1,631,247–1,631,416	3/3
HCN4	TSS/promoter-associated	chr15:73,367,019–73,367,274	chr15:73,366,973–73,367,328	chr15:73,366,999–73,367,212	3/3
GATA4	Promoter-associated	chr8:11,703,296–11,703,400	chr8:11,703,103–11,703,450	no peak called	2/3
TBX5	Intragenic regulatory	chr12:114,381,220–114,381,452	no peak called	chr12:114,381,218–114,381,440	2/3
NEG-2	Negative genomic reference	no peak called	no peak called	no peak called	0/3

Table S4b. Primers and amplicon coordinates

Locus	Amplicon (GRCh38)	Size (bp)	Forward primer (5′→3′)	Reverse primer (5′→3′)
MYOCD	chr17:12,665,845–12,665,924	80	TGGCAAAGTTGGGACCTTCA	GCGTTCCTGTCTGATGTCAT
TNNT2	chr1:201,377,982–201,378,106	125	AAAGCAGAGGCCAGCAACCCAG	ACTCTTGCCCTCTGCCTTCCAAGA
WNT5B	chr12:1,631,314–1,631,433	120	AACTGTCAGTCCCAGGGCACTG	ACCAGGAGTTGGCGTCTGTCAG
HCN4	chr15:73,367,109–73,367,191	83	GGCTGAATGACCCGGAGCT	CCTTAAGCCACATATTCCTCC
GATA4	chr8:11,703,269–11,703,397	129	ACGCGGTAATAGGGCCCTGTGATT	ATGTGTCAGGCCTGAGTTGGGAGT
TBX5	chr12:114,381,286–114,381,395	110	TCATCCCTCCTGCTTTTCTC	TGATAACAGCCAGGCAGGAA
NEG-2	chr5:21,300,013–21,300,117	105	ATCTCCTGTCTGTTCAGTGTG	CATAATGCAATAGGCGAAGGC

Footnotes to Table S4.

- All coordinates are GRCh38/hg38, 1-based and inclusive. Peak coordinates are reported for the MACS2 calls that overlap the corresponding amplicon; they are not amplicon coordinates.
- *MYOCD*: a peak was called at this locus in all three replicates, but in Hand1-3 the called peak boundary lies 65 bp distal to the amplicon and therefore does not overlap it.
- *GATA4*: the tested promoter-associated peak was supported by two of three replicates. The strongest *GATA4*-associated peak in the dataset was intragenic and was not tested.
- *TBX5*: the tested peak is intragenic (intronic) and was supported by two of three replicates.
- *WNT5B*: the tested peak is associated with the alternative transcript ENST00000542408 rather than with the canonical promoter.
- NEG-2: no peak was called within the region in any replicate; the nearest called peak lay 652 kb (Hand1-1), 182 kb (Hand1-2) and 2.3 Mb (Hand1-3) away. NEG-2 was used only as a within-sample ΔCt normalization reference and its selection was derived from the same CUT&Tag data; this does not constitute independent verification that the region is unoccupied.

Supplementary Table S5. Per-sample cell retention and quality metrics

A. Cell Ranger-called cells and cells retained in the final analysis

Sample	Cell Ranger-called cells	Final retained cells	Cumulative cells not retained	Cumulative not retained (%)
CON-1	11,181	9,800	1,381	12.35
CON-2	12,998	11,280	1,718	13.22
CON-3	11,806	10,430	1,376	11.66
KO-1	9,507	7,426	2,081	21.89
KO-2	14,139	12,230	1,909	13.50
KO-3	15,518	12,788	2,730	17.59
Total	75,149	63,954	11,195	14.90

B. Quality metrics of the final retained cells

Sample	Median UMI count	Median detected genes	Detected genes, P10–P90	Median mitochondrial (%)	P90 mitochondrial (%)
CON-1	8,365	3,144	794.9–5,226.1	1.03	3.38
CON-2	8,891	3,152	750–5,260.1	0.90	3.28
CON-3	8,162	2,966	797–4,877	0.83	3.21
KO-1	15,709.5	5,441	1,143.5–7,089	0.75	1.44
KO-2	5,429.5	2,868.5	973–5,725.2	0.70	1.46
KO-3	7,232.5	3,430	1,220–6,880	0.69	1.44

Notes. CON-1/KO-1, CON-2/KO-2 and CON-3/KO-3 are matched differentiation batches. The six samples contain 63,954 retained cells (31,510 CON and 32,444 HAND1-KO) across 24 retained clusters.

Cell Ranger-called counts were obtained from the original metrics_summary.csv files and verified against the corresponding filtered-matrix barcode lists. Every retained barcode was matched to its original sample. These initial counts represent called cells, not all sequenced droplets or absolute cell numbers in the organoids.

Cumulative cells not retained = Cell Ranger-called cells − final retained cells; the percentage uses Cell Ranger-called cells as the denominator. This difference combines all intervening exclusions and must not be interpreted as a doublet-specific exclusion count or rate. Step-specific filtering records and doublet-removal counts/rates were not recoverable (NR), rather than zero. All retained cells carry the inherited Singlet label, which does not quantify the cells excluded earlier.

UMI medians are from the existing raw-count-based quality audit; detected-gene and mitochondrial-transcript metrics were verified against the retained-cell metadata. P10 and P90 denote the 10th and 90th percentiles. Quality distributions are descriptive and may reflect both cell composition and technical factors. No additional filtering was applied for these tables.

Supplementary Table S6. Sample-level cell composition and paired changes

A. Retained-cell counts and percentages by sample

Annotation category	CON-1	CON-2	CON-3	KO-1	KO-2	KO-3
Cardiomyocytes	4,550 (46.43)	5,502 (48.78)	4,665 (44.73)	262 (3.53)	262 (2.14)	240 (1.88)
Epicardial-like cells	1,052 (10.73)	1,055 (9.35)	1,102 (10.57)	265 (3.57)	424 (3.47)	400 (3.13)
Cardiac progenitor-like cells	1,701 (17.36)	1,720 (15.25)	1,910 (18.31)	908 (12.23)	1,546 (12.64)	1,484 (11.60)
Mesodermal/ mesenchymal-like cells	156 (1.59)	127 (1.13)	173 (1.66)	4,382 (59.01)	7,308 (59.75)	8,355 (65.33)
Endothelial cells	413 (4.21)	396 (3.51)	381 (3.65)	54 (0.73)	148 (1.21)	107 (0.84)
Endodermal-like cells	141 (1.44)	386 (3.42)	252 (2.42)	66 (0.89)	135 (1.10)	71 (0.56)
Epithelial cells	999 (10.19)	1,117 (9.90)	1,034 (9.91)	429 (5.78)	810 (6.62)	587 (4.59)
hPSC-like cells	460 (4.69)	588 (5.21)	597 (5.72)	508 (6.84)	598 (4.89)	578 (4.52)
Cycling cells	276 (2.82)	345 (3.06)	261 (2.50)	412 (5.55)	334 (2.73)	519 (4.06)
Low-complexity cells	52 (0.53)	44 (0.39)	55 (0.53)	140 (1.89)	665 (5.44)	447 (3.50)
Total retained cells	9,800 (100.00)	11,280 (100.00)	10,430 (100.00)	7,426 (100.00)	12,230 (100.00)	12,788 (100.00)

Entries are cell counts (percentages). Each percentage uses all retained cells in that sample as the denominator; rounded values may not sum to exactly 100%. No categories were omitted, including the Cycling and Low-complexity categories.

B. Equal-weight summaries of the three samples per condition

Annotation category	CON proportion (%), mean ± SD	HAND1-KO proportion (%), mean ± SD
Cardiomyocytes	46.64 ± 2.03	2.52 ± 0.89
Epicardial-like cells	10.22 ± 0.75	3.39 ± 0.23
Cardiac progenitor-like cells	16.97 ± 1.57	12.16 ± 0.52
Mesodermal/mesenchymal-like cells	1.46 ± 0.29	61.37 ± 3.46
Endothelial cells	3.79 ± 0.37	0.92 ± 0.25
Endodermal-like cells	2.43 ± 0.99	0.85 ± 0.28
Epithelial cells	10.00 ± 0.17	5.66 ± 1.02
hPSC-like cells	5.21 ± 0.52	5.42 ± 1.25
Cycling cells	2.79 ± 0.28	4.11 ± 1.41
Low-complexity cells	0.48 ± 0.08	3.61 ± 1.78

Each differentiation sample receives equal weight; cells are not pooled to calculate group means. SD denotes the sample standard deviation across the three differentiation batches, not SEM or variability among individual cells.

Supplementary Table S6. Continued

C. Paired differences and exploratory sample-level tests

Annotation category	Pair 1 Δ (pp)	Pair 2 Δ (pp)	Pair 3 Δ (pp)	Mean Δ (pp)	Observed Δ range (pp)	Exact *P*	Holm *P*
Cardiomyocytes	−42.90	−46.63	−42.85	−44.13	−46.63 to −42.85	0.250	1.000
Epicardial-like cells	−7.17	−5.89	−7.44	−6.83	−7.44 to −5.89	0.250	1.000
Cardiac progenitor-like cells	−5.13	−2.61	−6.71	−4.81	−6.71 to −2.61	0.250	1.000
Mesodermal/ mesenchymal-like cells	+57.42	+58.63	+63.68	+59.91	+57.42 to +63.68	0.250	1.000
Endothelial cells	−3.49	−2.30	−2.82	−2.87	−3.49 to −2.30	0.250	1.000
Endodermal-like cells	−0.55	−2.32	−1.86	−1.58	−2.32 to −0.55	0.250	1.000
Epithelial cells	−4.42	−3.28	−5.32	−4.34	−5.32 to −3.28	0.250	1.000
hPSC-like cells	+2.15	−0.32	−1.20	+0.21	−1.20 to +2.15	1.000	1.000
Cycling cells	+2.73	−0.33	+1.56	+1.32	−0.33 to +2.73	0.500	1.000
Low-complexity cells	+1.35	+5.05	+2.97	+3.12	+1.35 to +5.05	0.250	1.000

Δ = HAND1-KO minus CON, in percentage points (pp), calculated within each matched differentiation batch. The mean paired difference is the effect-size estimate and test statistic. The range is the observed minimum–maximum across the three pairs, not a confidence interval.

Exploratory analysis. For each of the 10 annotation categories, a two-sided exact paired sign-flip test enumerated all $2^3 = 8$ possible sign assignments to the three paired differences. *P* equals the fraction of assignments with an absolute mean difference at least as large as the observed value, including ties. Holm adjustment was applied across all 10 categories to control the family-wise error rate without assuming independence between categories. Calculations used unrounded counts and proportions.

Assumptions and scope. The independent units are the three differentiation batches, not individual cells. The exact sign-flip interpretation assumes exchangeability of condition labels within pairs under the null, or equivalently sign symmetry of independent paired differences. This assumption cannot be established from only three pairs. These are exploratory comparisons in the studied clone pair; repeated differentiations do not provide independent KO-clone replication. No cells or samples were excluded for this supplementary analysis.

With three nonzero paired differences, the minimum attainable two-sided *P* is 0.25. No category reaches $P < 0.05$ in this test, before or after adjustment. This limited resolution does not demonstrate equivalence or absence of an effect. Individual paired differences and their observed range are reported rather than imposing a distributional model to obtain confidence intervals from only three pairs.

The annotation categories match the publication metadata. Cardiomyocytes comprise clusters 0, 7, 12, 13 and 25; mesodermal/mesenchymal-like cells comprise clusters 1, 3, 4, 15, 17 and 24. The latter is broader than the focused KO-enriched target region used in the marker analysis. Cycling and Low-complexity are state/quality annotations, not distinct lineage identities. Relative composition does not establish absolute cell expansion, lineage conversion or the absence of differential recovery bias.
