# Supplementary material for: Human Cardiac Organoids Reveal HAND1 Loss-Associated Alterations in Cell Composition During Early Cardiac Differentiation
Source: Cells. 2026 Oct 26;15(21):1948. doi: 10.3390/cells15211948 (PMC13761964; doi:10.3390/cells15211948)
Supplement: Supplementary file 1 [file cells-15-01948-s001.zip › Supplementary_Video.pdf]

Thalidomide

Thalidomide_control.avi   Thalidomide_0.01ug_ml.avi

Supplementary Video S1. Representative video of calcium transient activity in cardiac organoids exposed to thalidomide. Cardiac organoids were continuously exposed to thalidomide from the initiation of differentiation. On day 9 of differentiation, calcium transient imaging was performed using Fluo-4 AM. The video shows representative calcium signal activity in organoids from the vehicle control group and the 0.01 μg/mL thalidomide-treated group.
